# Low Socioeconomic Status Is Associated with Worse Survival in Children with Cancer: A Systematic Review

**DOI:** 10.1371/journal.pone.0089482

**Published:** 2014-02-26

**Authors:** Sumit Gupta, Marta Wilejto, Jason D. Pole, Astrid Guttmann, Lillian Sung

**Affiliations:** 1 Division of Haematology/Oncology, the Hospital for Sick Children, Toronto, Ontario, Canada; 2 Department of Paediatrics, University of Toronto, Toronto, Ontario, Canada; 3 Pediatric Oncology Group of Ontario, Toronto, Ontario, Canada; 4 Institute for Clinical and Evaluative Sciences, Toronto, Ontario, Canada; 5 Division of Paediatric Medicine, Hospital for Sick Children, Toronto, Ontario, Canada; 6 Program in Child Health Evaluative Sciences, the Hospital for Sick Children, Toronto, Ontario, Canada; 7 Institute for Health, Policy Management and Evaluation, University of Toronto, Toronto, Ontario, Canada; University of Pennsylvania, United States of America

## Abstract

**Background:**

While low socioeconomic status (SES) has been associated with inferior cancer outcome among adults, its impact in pediatric oncology is unclear. Our objective was therefore to conduct a systematic review to determine the impact of SES upon outcome in children with cancer.

**Methods:**

We searched Ovid Medline, EMBASE and CINAHL from inception to December 2012. Studies for which survival-related outcomes were reported by socioeconomic subgroups were eligible for inclusion. Two reviewers independently assessed articles and extracted data. Given anticipated heterogeneity, no quantitative meta-analyses were planned *a priori.*

**Results:**

Of 7,737 publications, 527 in ten languages met criteria for full review; 36 studies met final inclusion criteria. In low- and middle-income countries (LMIC), lower SES was uniformly associated with inferior survival, regardless of the measure chosen. The majority of associations were statistically significant. Of 52 associations between socioeconomic variables and outcome among high-income country (HIC) children, 38 (73.1%) found low SES to be associated with worse survival, 15 of which were statistically significant. Of the remaining 14 (no association or high SES associated with worse survival), only one was statistically significant. Both HIC studies examining the effect of insurance found uninsured status to be statistically associated with inferior survival.

**Conclusions:**

Socioeconomic gradients in which low SES is associated with inferior childhood cancer survival are ubiquitous in LMIC and common in HIC. Future studies should elucidate mechanisms underlying these gradients, allowing the design of interventions mediating socioeconomic effects. Targeting the effect of low SES will allow for further improvements in childhood cancer survival.

## Introduction

Socioeconomic status (SES), a multi-dimensional construct encompassing economic resources, power and social standing, has been associated with a number of health outcomes.[1]–[4] Understanding the mechanisms behind such associations is necessary in order to reduce health disparities. Among adult patients, strong evidence exists supporting socioeconomic gradients in cancer mortality. [5].

By contrast, the equivalent pediatric literature is sparse and predominantly restricted to low- and middle-income countries (LMIC). [6], [7] High-income country (HIC) studies have yielded seemingly contradictory results.[8]–[10] Given differences in cure rates and developmental position, adult socioeconomic gradients cannot be extrapolated to children with cancer.

We therefore undertook the first systematic review of the literature examining the impact of SES upon pediatric oncology outcomes. Our primary objective was to determine the impact of income- and education-based measures of SES on event-free survival (EFS), overall survival (OS) and disease-free survival (DFS) among children with cancer. Secondary objectives included determining the effect of other SES measures, as well as the effect of SES on treatment-related mortality (TRM), relapse and abandonment of therapy.

## Methods

The conduct of the review followed the PRISMA framework. [11] Both the PRISMA Checklist and the initial protocol can be found in Checklist S1 and Text S1.

### Data Sources

We performed electronic searches of Ovid Medline, EMBASE and CINAHL from inception to December 10^th^, 2012 with the assistance of a library scientist. The Medline search strategy is illustrated in Table 1, with complete strategies illustrated in Text S2.

**Table 1:** Medline Search Strategy.

Set	History	Results	Comments
1	“emigration and immigration”/or residence characteristics/or “catchment area (health)”/or housing/or public housing/or health status disparities/or Healthcare Disparities/or ruralhealth services/or suburban health services/or urban health services/or exp Insurance/orexp Health Services Accessibility/or exp Socioeconomic Factors/	54,3627	SES Terms
2	Exp Neoplasms/	2,416,057	Neoplasm terms
3	1 and 2	3,227,924	Base clinical set
4	limit 3 to “all child (0 to 18 years)”	4,042	Age group limit
5	(infan* or child* or adolescen* or youth* orteen* or pediatric* or paediatric*).mp.	2,961,284	Age group textword terms
6	4 or (3 and 5)	4,533	FINAL Results

### Study Selection

Inclusion and exclusion criteria were defined *a priori*. Inclusion criteria were: (1) ecologic, cross-sectional, cohort, case-control or randomized control trial designs; (2) pediatric data available, with pediatric ages defined by authors, and (3) at least one pre-specified survival-related outcome reported by subgroups defined by a pre-specified socioeconomic variable (see below). Biologic factors may account for a portion of the disparities in outcome seen between different ethnic groups. [12] Since the independent effects of biology and SES cannot be disentangled when ethnicity is the sole proxy of SES, such studies were excluded. There was no restriction by language. Two reviewers (SG, MW) independently evaluated identified titles and abstracts, retrieved any potentially relevant manuscript and determined eligibility; discrepancies were resolved through consensus. Agreement between reviewers was assessed using the kappa statistic. [13] Non-English articles were assessed with the assistance of pediatric oncologists whom were native speakers of the relevant language.

### Data Abstraction

Two reviewers (SG, MW) independently abstracted data using standardized forms. The primary outcomes were EFS, OS and DFS; secondary outcomes were specific causes of treatment failure (TRM, relapse, abandonment). Relative survival was assumed to be comparable to OS. Multiple measures of SES exist in the literature, reflecting three main domains: material resources, knowledge related assets and social standing. [14] Though income and education (including measures of occupation) were the key variables of interest in this study, we included a broad range of SES measures reflecting these domains: material possession (e.g. car ownership), family composition (e.g. marital status), health insurance status, health care accessibility and immigrant status. Both ecologic and individual-level variables were included. When measures over multiple time periods were available, only the most contemporaneous time period was recorded. Study authors were contacted to solicit missing data.

Study quality was assessed using a framework of potential biases developed by Hayden et?al to evaluate prognosis studies. [15] Four key indicators of study quality relevant for studies examining the impact of SES were identified *a priori*: (1) the degree to which study samples reflected underlying populations, (2) whether loss to follow-up was associated with socioeconomic characteristics, (3) whether potential confounders were accounted for and (4) the appropriateness of the analysis. Further details are provided in the online supplemental data. When assessing the degree to which study samples represented the general population, samples derived from clinical trials were judged to be only partly representative of the overall population, as patients of low SES who consent to trials may be systematically different than those who do not. [16], [17] Single institution studies were also assessed as only partly representative. The loss to follow-up quality indicator was judged not applicable for settings in which abandonment of therapy constituted a significant cause of treatment failure. [18] As various indicators measure different domains of socioeconomic position, accounting for confounding was assessed as adequate if both a measure of disease risk and a second SES indicator were included. Analyses that were not based on time-to-event data were assessed as partially adequate.

### Analysis

Given the anticipated heterogeneity in settings, SES measures and malignancies, no quantitative meta-analyses were planned. The magnitude and underlying mechanisms of any association between SES and outcome are likely to differ between developing and developed countries. The results were therefore summarized separately for LMIC and for HIC, as defined by the World Bank using Gross National Income per capita (LMIC <$12,616 vs. HIC ≥$12,616). [19].

As the unit of analysis varied markedly even among studies investigating a common SES variable (e.g. per unit of monthly income vs. per income quintile), we could not compare magnitudes of association across studies. Consequently, measures of association between SES and outcome were plotted on a single graph in which sample size was represented on the x-axis. Positive associations (defined as higher SES associated with better outcome) were placed to the right of the y-axis while negative associations (defined as higher SES associated with worse outcome) were placed to the left, regardless of statistical significance or magnitude. Points more distal from the y-axis therefore do not represent greater degrees of association. When the SES measure was categorical (e.g. income quintiles), the direction of the association was determined by comparing outcomes between the highest and lowest SES categories. For each study, associations for only the highest aggregation of cancers were presented. Statistically significant associations were displayed in red and non-significant associations in black.

For studies describing the effect of dichotomous measures of income or insurance upon EFS, OS or DFS in acute lymphoblastic leukemia (ALL) or Hodgkin lymphoma (HL), the proportion of adverse outcomes attributable to low SES (attributable risk) was calculated by the following formula (p_e_ = proportion of the population exposed to the adverse prognosticator; RR = ratio of the cumulative incidence of adverse outcome in the two groups): [20] 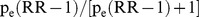 ALL and HL were chosen as they account for a significant percentage of incident cases of childhood cancer. The concept of attributable risk assumes that the relationship is causal and that no significant bias or confounding exists. Attributable risks were also calculated for recently discovered biologic prognosticators as comparators. These prognosticators were chosen by the authors based on their prominence in either clinical practice (e.g. minimally residual disease) or laboratory research (e.g. CRLF2 expression).

### Ethics Statement

Institutional review board approval was not required as only group-level, and not individual-level data were obtained from already published studies.

## Results

**Figure 1:**
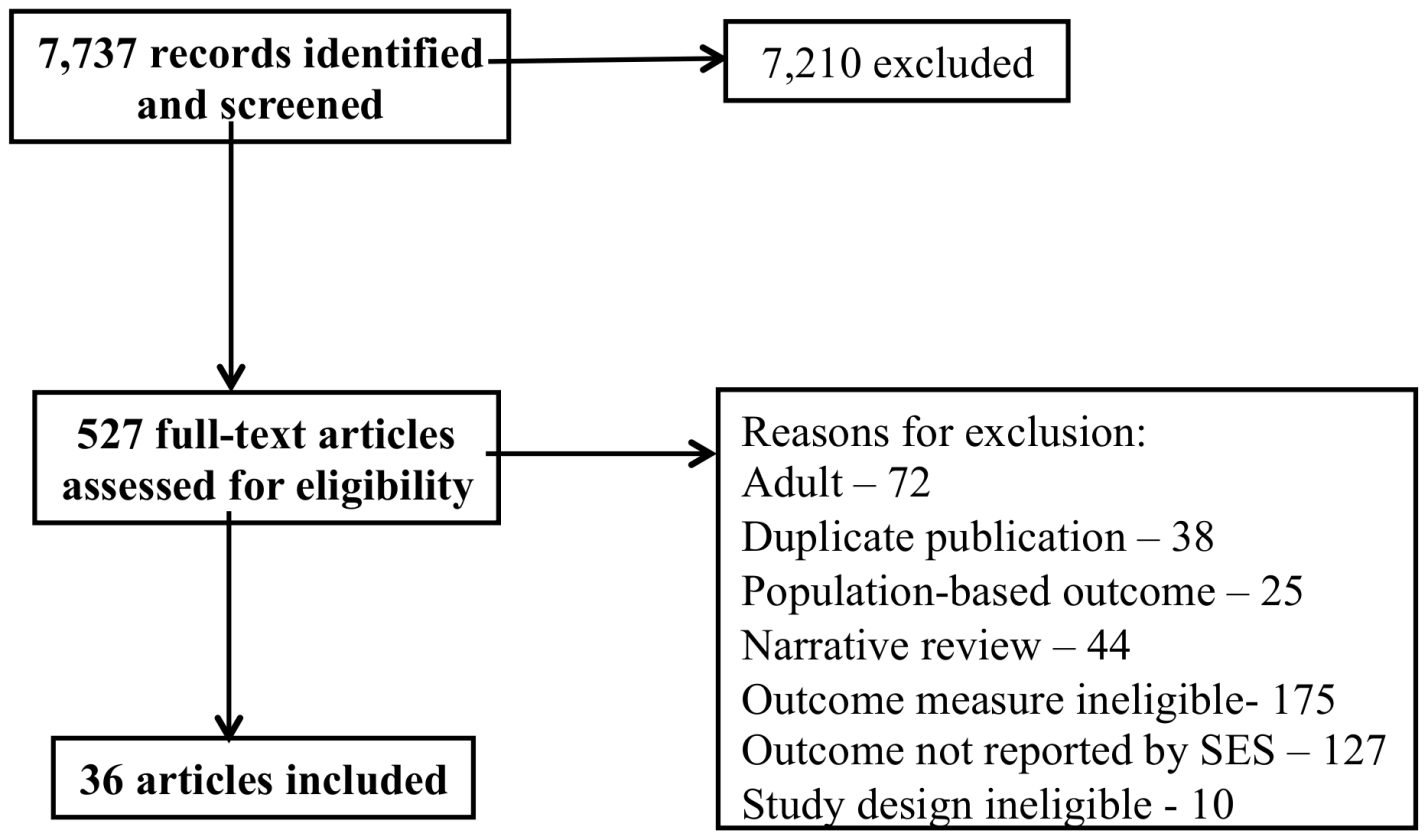
PRISMA flow diagram.

Figure 1 illustrates the flow of study identification and selection. A total of 7,737 abstracts were identified by the search strategy; 527 articles in ten languages were retrieved for full evaluation. Of these, 36 met eligibility criteria. The kappa statistic of agreement between the two reviewers was 0.82 (95% confidence interval (CI) 0.72–0.91). Characteristics of the included studies, including indicators of study quality, are shown in Table 2. Though most studies were of acceptable quality, only half accounted for potential confounders.

**Table 2:** Characteristics of included studies.

Characteristic	Studies, N (%)	
	LMIC (N = 10)	HIC (N = 26)
Malignancy		
All cancers	0 (0.0)	8 (30.8)
Leukemia or lymphoma	9 (90.0)	15 (57.7)
Solid tumor	1 (10.0)	1 (3.8)
Central nervous system tumor	0 (0.0)	2 (7.7)
Type of socioeconomic variable examined		
Ecologic	1 (10.0)	13 (50.0)
Income-based	7 (70.0)	2 (7.7)
Education-based^a^	6 (60.0)	10 (38.5)
Other^b^	5 (50.0)	10 (38.5)
Sample Size		
<100	1 (10.0)	4 (15.4)
100**–**999	9 (90.0)	9 (34.6)
1,000**–**9,999	0 (0.0)	12 (46.2)
≥10,000	0 (0.0)	1 (3.8)
Restricted to adolescents/young adults^c^		
Yes	0 (0.0)	2 (7.7)
No	10 (100.0)	24 (92.3)
Study sample adequately reflective of general population^d^		
Yes	8 (80.0)	21 (80.7)
No/Partial/Unsure	2 (20.0)	5 (19.2)
Loss to follow-up unrelated to socioeconomic status^d^		
Yes	3 (30.0)	21 (80.7)
No/Partial/Unsure	1 (10.0)	5 (19.2)
Not applicable	6 (60.0)	0 (0.0)
Potential confounders accounted for^d^		
Yes	6 (60.0)	12 (46.2)
No/Partial/Unsure	4 (40.0)	14 (53.8)
Analysis appropriate^d^		
Yes	8 (80.0)	18 (69.2)
No/Partial/Unsure	2 (20.0)	8 (30.8)

HIC – high-income countries; LMIC – low- and middle-income countries.

a Also included occupation-based measures of socioeconomic status.

b Included measures of material possession, family composition, insurance status, immigrant status, and health care accessibility.

c As defined by study authors.

d See supplemental data for definitions of study quality variables.

### Low- and Middle-income Country Studies

The results of the ten eligible LMIC studies are shown in Table 3, with full details available in Table S1. Of the ten, seven found at least one measure of low SES to be significantly associated with inferior outcome.[21]–[27] The remaining three found no significant association.[28]–[30] When restricted to studies examining the primary outcomes of EFS, OS or DFS, 6/7 (85.8%) studies showed at least one statistically significant association where lower SES was associated with worse survival.

**Table 3:** Eligible studies examining the impact of socioeconomic status upon outcome in children with cancer in low- and middle-income countries.

	Country	Malignancy	N	OutcomeMeasure	Ecologic Measures	Income Measures	Education Measures^a^	Other SES Measures
Bonilla 2010	El Salvador	Standard risk ALL	260	EFS	**–**	**HR 0.84; Per $100** **increase**	**HR 0.49; ≥Secondary vs.** **≤primary**	Telephone ownership NS
**–**	**–**	**–**	**–**	**–**	**–**	**–**	**–**	Mode of transport NS
**–**	**–**	High risk ALL	183	EFS	**–**	Monthlyincome NS	Parentaleducation NS	Telephone ownership NS
**–**	**–**	**–**	**–**	**–**	**–**	**–**	**–**	Mode of transport NS
Mostert 2010	Indonesia	ALL	283	EFS	**–**	**HR 2.6; 2nd/3rd class ward vs.** **VIP/1st class ward, based on income**	**.**	**.**
Tang 2008	China	ALL	346	EFS	**–**	**.**	**–**	**5-year EFS 61.2% urban vs. 30.3%** **rural; p<0.0001** c
Dinand 2007	India	Hodgkin Lymphoma	145	EFS	**–**	**HR 5.4; Low vs.** **high Kuppuswami score** b		**–**
Pedrosa 2007	Brazil	Non-Hodgkin Lymphoma	110	OS	**–**	Family income NS	Maternal education NS	**–**
Carlos 2002	Mexico	Retinoblastoma	552	OS	**HR 2.38; Most marginalized vs. least**	**–**	**–**	**–**
Viana 1998	Brazil	ALL	167	DFS	**–**	**5-year DFS 58% for those >0.4 ×** **minimum wage vs. 8% for those <0.4 ×** **minimum wage; p<0.0001**	**–**	**>4 kw hours daily electric consumption vs.** **<4 kw hours; p = 0.0003**
**–**	**–**	**–**	**–**	**–**	**–**	**–**	**–**	**Very poor vs. fair-good housing conditions; p = 0.006**
Gupta 2009	El Salvador	AML	78	TRM	**–**	Monthly income NS	Parental education NS	Telephone ownership NS
**–**	**–**	**–**	**–**	**–**	**–**	**–**	**–**	Number of family members NS
**–**	**–**	**–**	**–**	**–**	**–**	**–**	**–**	Cost to travel to clinic NS
Wang 2011	China	ALL	323	Abandonment	**–**	**–**	Paternal education NS	**32.5% abandonment good housing conditions** **vs. 83.3% poor; p<0.001**
**–**	**–**	**–**	**–**	**–**	**–**	**–**	Maternal education NS	**–**
Kulkarni 2010	India	ALL	532	Abandonment	**–**	Kuppuswami score NS^b^	**–**	**–**

ALL – acute lymphoblastic leukemia; AML – acute myeloid leukemia; DFS – disease free survival; EFS – event free survival; HR – hazard ratio; N – number; NS – non-significant; OS – overall survival; SES – socioeconomic status; TRM – treatment related mortality.

Bolded variables indicate statistically significant associations. Magnitudes of non-significant associations and confidence intervals of significant associations can be found in Table S1, along with definitions of each variable.

a Education measures also include occupation-based measures.

b Aggregate score based on income, education and occupation.

c Urban residents also had medical insurance while rural residents did not.

**Figure 2:**
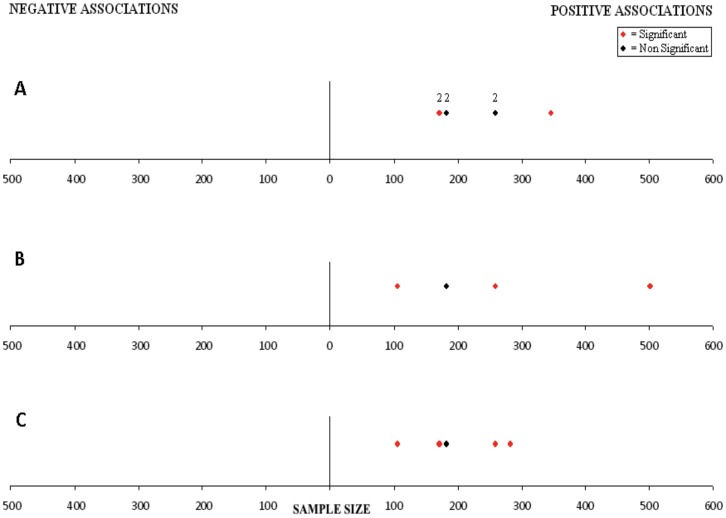
Associations between socioeconomic measures and event-free and overall survival in low- and middle-income countries. A. Measures of material possession, family composition, insurance status, immigrant status, and health care accessibility. B. Measures of education and occupation. C. Measures of income. Positive = lower socioeconomic status associated with inferior outcome; Negative = lower socioeconomic status associated with superior outcome. Magnitudes of association are not plotted. Statistically significance is denoted in red. Data points with a number above represent multiple socioeconomic variables.

Figure 2 illustrates each association between a socioeconomic variable and outcome plotted by study sample size, restricted to LMIC studies examining EFS, OS or DFS. One Brazilian study of non-Hodgkin lymphoma provided log rank p values of without information on the directions of association; none of these were statistically significant. [30] Regardless of the SES measure chosen, lower SES was always associated with inferior EFS/OS/DFS, with the majority of associations statistically significant. There were no studies that showed that lower SES was associated with better survival irrespective of statistical significance.

### High-income Country Studies

The results of the 26 eligible studies conducted in HIC are shown in Table 4, all of which used EFS or OS as their outcome. Full details are available in Table S2. Individual-level and ecologic measures of SES were used by 13 (50.0%) and 10 (38.5%) studies respectively; three studies (11.5%) used both. Of the 26, 14 (53.8%) showed at least one measure of low SES to be significantly associated with inferior outcome.[10], [31]–[43].

**Table 4:** Eligible studies examining the impact of socioeconomic status upon outcome in children with cancer in high-income countries.

	Country	Outcome Measure	Malignancy	N	Ecologic Measures	Income Measures	Education Measures^a^	Other SES Measures
Metzger 2008	USA	EFS	Hodgkin lymphoma	327	**HR 1.9; High** **poverty county** **vs. low**	–	**–**	–
Bhatia 2002	USA, Canada	EFS	ALL	1596	–	Annual household income NS	Paternal educationNS	–
–	–	–	–	–	–	–	Maternal educationNS	–
Hann 1981	England	5 year EFS	ALL	209	–	–	Paternal occupationNS	–
Lightfoot 2012	England, Scotland, Wales	OS	ALL	1559	**HR 1.29;** **Deprived vs.** **affluent**	–	Paternal occupationNS	–
Syse 2012	Norway	OS	Cancers	6280	–	Household income NS	**OR 1.2; ≤High** **school vs. ≥College**	Marital status NS
–	–	–	–	–	–	**–**	–	Number of children NS
Rondelli 2011	Italy	OS	ALL	3522	–	**.**	–	**HR 1.70; Immigrant** **vs. non-immigrant**
Walsh 2011^b^	Ireland	5 year OS	All Cancers	1440	SAHRUdeprivationindexNS	–	–	–
Youlden 2011	Australia	5 year OS	Cancers	6289	Disadvantageindex NS	–	–	**HR 1.55; Remote** **vs. Major city**
Crouch 2009^c^	UK	5 year OS	All cancers	654	**Affluent 70% OS to** **deprived 64%;** **trend p<0.5**	–	–	–
Hsieh 2009	USA	OS	NB	1777	**–**	**–**	**–**	**5-year OS Urban** **county 63% OS vs.** **rural county 55%; p = 0.04**
Kent 2009	USA	OS	Leukemias	4158	Census-baseddeprivationindex NS	–	–	**HR 1.56; Any** **insurance vs.** **none/unknown**
Birch 2008^b^^,^ c	England	5 year OS	All Cancers	31722	**Affluent 71% to deprived 70%;** **trend p = 0.001**	–	–	–
Moschovi 2007	Greece	OS	MB	50	–	–	Maternal education NS	Place of residence NS
Perez-Martinez 2007^d^	Spain	5 year OS	All cancers	90+	–	.	–	Immigrant status NS
Tseng 2006	England, Wales	5 year OS	Malignant CNS	3169	Carstairsindex NS	–	–	–
Charalampopolou 2004	Greece	OS	ALL	293	–	–	Maternaleducation NS	**HR 2.85; Other vs.** **married**
–	–	–	–	–	–	–	–	**HR 0.63;** **Per child**
Coleman 1999	England, Wales	5 year OS	Hodgkin lymphoma	189	Carstairsindex NS	–	–	–
–	–	–	NHL	273	Carstairsindex NS	–	–	–
–	–	–	CNS	1050	Carstairsindex NS	–	–	–
–	–	–	Wilms	257	Carstairsindex NS	–	–	–
–	–	–	OST	117	Carstairsindex NS	–	–	–
–	–	–	ES	97	Carstairsindex NS	–	–	–
–	–	–	STS	319	Carstairsindex NS	–	–	–
–	–	–	GCT	121	Carstairsindex NS	–	–	–
McKinney 1999^e^	UK	OS	All Cancers	1979	Carstairsindex NS	–	–	–
Schillinger 1999	England, Wales	5 year OS	ALL	5566	Carstairsindex NS	–	–	–
Coebergh 1996	Netherlands	5 year OS	Standard-risk ALL	367	–	–	Parentaleducation NS	–
–	–	–	High-risk ALL	141	–	–	Parentaleducation NS	–
–	–	–	AML	67	–	–	Parentaleducation NS	–
Hord 1996	USA	5 year OS	ALL	178	–	–	–	**OR 0.61; Total insurance coverage vs. at least partially uncovered**
Petridou 1994	Greece	OS	Leukemias	120	–	.	Paternaloccupation NS	**HR 0.29; Private car vs. none**
–	–	–	–	–	–	–	Paternaleducation NS	Maternity hospital type NS
–	–	–	–	–	–	–	Maternaleducation NS	Ability to choose doctor NS
McWhirter 1983	Australia	5 year OS	ALL	70	–	–	**High social class 59%** **OS vs. low 27%**	–
Szklo 1978	USA	2 year OS	ALL	55	**High rental** **value 51% OS** **vs. low rental** **value 28%; p<0.005**	–	–	–
Byrne 2011	USA	Medianduration	AML (Age 0–9)	84	Communitypoverty level NS	–	–	–
–	–		AML (Age 10–19)	102	Communitypoverty level NS	–	–	–
Walters 1972^f^	USA	Medianduration	ALL	334	–	–	**16.2 months lowest SES** **vs. 24.3 months** **highest**	–

ALL – acute lymphoblastic leukemia; AML – acute myeloid leukemia; CNS – central nervous system tumors; EFS – event free survival; ES – Ewing sarcoma; GCT – germ cell tumors; HR – hazard ratio; LR – log rank; MB – medulloblastoma; N – number; NB – neuroblastoma; NHL – non-Hodgkin lymphoma; OR – odds ratio; OS – overall survival; OST – osteosarcoma; RR – relative risk; SES – socioeconomic status; STS – soft tissue sarcoma; UK – United Kingdom; USA – United States of America.

Bolded variables indicate statistically significant associations. Magnitudes of non-significant associations and confidence intervals of significant associations can be found in Table S2, along with definitions of each variable.

a Education measures also include occupation-based measures.

b Individual malignancies within the overall category showed no significant association between SES and outcome.

c Adolescent and young adult population.

d Immigrant patients from one center were compared to a historical control.

e Within the overall malignancy category, leukemias did show a significant association between lower SES and inferior outcome.

f No statistical analysis was presented, though the authors state that survival was “directly related to SES”.

**Figure 3:**
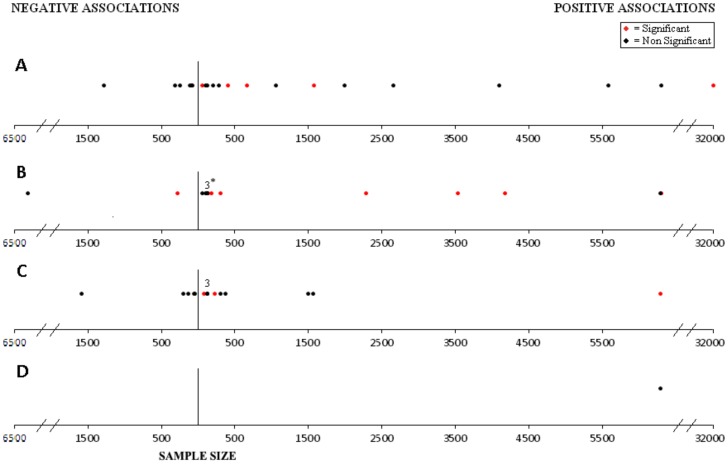
Associations between socioeconomic measures and event-free and overall survival in high-income countries. A. Ecologic measures B. Measures of material possession, family composition, insurance status, immigrant status, and health care accessibility. C. Measures of education and occupation. D. Measures of income. Positive = lower socioeconomic status associated with inferior outcome; Negative = lower socioeconomic status associated with superior outcome. Magnitudes of association are not plotted. Statistically significance is denoted in red. Data points with a number above represent multiple socioeconomic variables. 3* indicates 2 non-significant associations and one significant association.

Figure 3 illustrates each HIC association plotted by the study sample size. Of the 21 measures of association between ecologic SES variables and outcome, 15 (71.4%) showed lower SES to be associated with worse survival, five of which were statistically significant. The remaining six (28.6%) showed that lower SES was associated with superior outcome, none of which were statistically significant.

Of the 15 measures of association between individual parental education and outcome, ten (66.7%) showed that lower parental education was associated with worse survival, three of which were statistically significant. None of the five (38.5%) associations in which higher parental education was associated with worse survival were statistically significant.

Two studies examined the impact of family income. In one study, there was no association between annual income categorized above and below $30,000 and EFS (HR = 1.0). [44] The second study found that lower income was associated with worse OS though the association was not statistically significant. [42].

Of the 14 associations between the remaining individual-level SES variables and outcome, 12 (85.7%) showed that worse SES was associated with inferior outcome, seven of which were statistically significant. Two (14.3%) studies showed that better SES was associated with worse outcome. One of these two was statistically significant; among children with ALL in Greece, a higher number of siblings was associated with a lower risk of death (HR 0.63 per child; 95% CI 0.40–0.99). [10].

Figure S1 illustrates all associations between SES measures (individual or ecologic) and outcome from the subset of HIC studies conducted in the United States. Of eleven associations, eight (72.7%) showed that lower SES was associated with worse outcome; two were statistically significant. There were three associations in which better SES was associated with worse survival; none were statistically significant.

### Attributable Risk

Table 5 shows the proportion of adverse outcomes attributable to low socioeconomic measures of income or insurance as calculated from LMIC and HIC studies. Based on the selected studies, and assuming both causality and the absence of significant bias or confounding, eliminating the adverse effect of low socioeconomic status would result in a theoretical 22.9% to 74.8% reduction in adverse outcome among LMIC children. Among HIC children, 0.0% to 31.9% of adverse outcomes could be avoided.

**Table 5:** Proportion of adverse outcomes (attributable risk) due to poor socioeconomic prognosticators in studies of the effect of dichotomous measures of income and insurance in acute lymphoblastic leukemia and Hodgkin lymphoma, as well as of selected biologic prognosticators by way of comparison.

	Malignancy	Country	Category	Adverse Prognosticator	p_e_	RR	AR
Dinand 2007	HL	India	LMIC	Low SES, based on aggregate score including income	0.67	5.4	74.8%
Mostert 2010	ALL	Brazil	LMIC	Monthly per capita income <0.4 ×minimum wage	0.25	1.2	22.9%
Viana 1998	ALL	Indonesia	LMIC	2nd/3rd class ward, based on income	0.76	2.6	55.0%
Tang 2008	ALL	China	LMIC	Rural residence/no insurance	0.74	1.8	37.1%
Bhatia 2002	ALL	USA, Canada	HIC	Annual household income <$30,000	0.56	1.0	0.0%
Hord 1996	ALL	USA	HIC	At least partially uncovered by insurance	0.29	1.6	15.7%
Lightfoot 2012	ALL	England, Scotland, Wales	HIC	Deprived area, based in part on income	0.39	1.3	10.2%
Metzger 2008	HL	USA	HIC	County with high % children in poverty	0.52	1.9	31.9%
Borowitz 2008	SR-ALL	Multiple	HIC	MRD>0.01%	0.14	7.2	45.6%
Borowitz 2008	HR-ALL	Multiple	HIC	MRD>0.01%	0.30	3.2	39.4%
Loken 2012	AML	Multiple	HIC	Residual disease by flow cytometry	0.22	2.17	20.5%
Chen 2012	ALL	Multiple	HIC	High CRLF2 expression	0.18	1.86	13.1%

ALL – acute lymphoblastic leukemia; AML – acute myeloid leukemia; AR – attributable risk; HIC – high-income country; HL – Hodgkin lymphoma; LMIC – low- to middle-income country; MRD – minimal residual disease; p_e_ – proportion of population exposed to the adverse prognosticator; RR – risk ratio; SES – socioeconomic status.

## Discussion

In this systematic review, we found that among children with cancer in LMIC, measures of low SES were uniformly associated with inferior outcome. The majority of these associations were statistically significant. The results in HIC were less uniform although the majority of associations (including all but one of the statistically significant associations) also linked lower SES and worse outcome.

**Figure 4:**
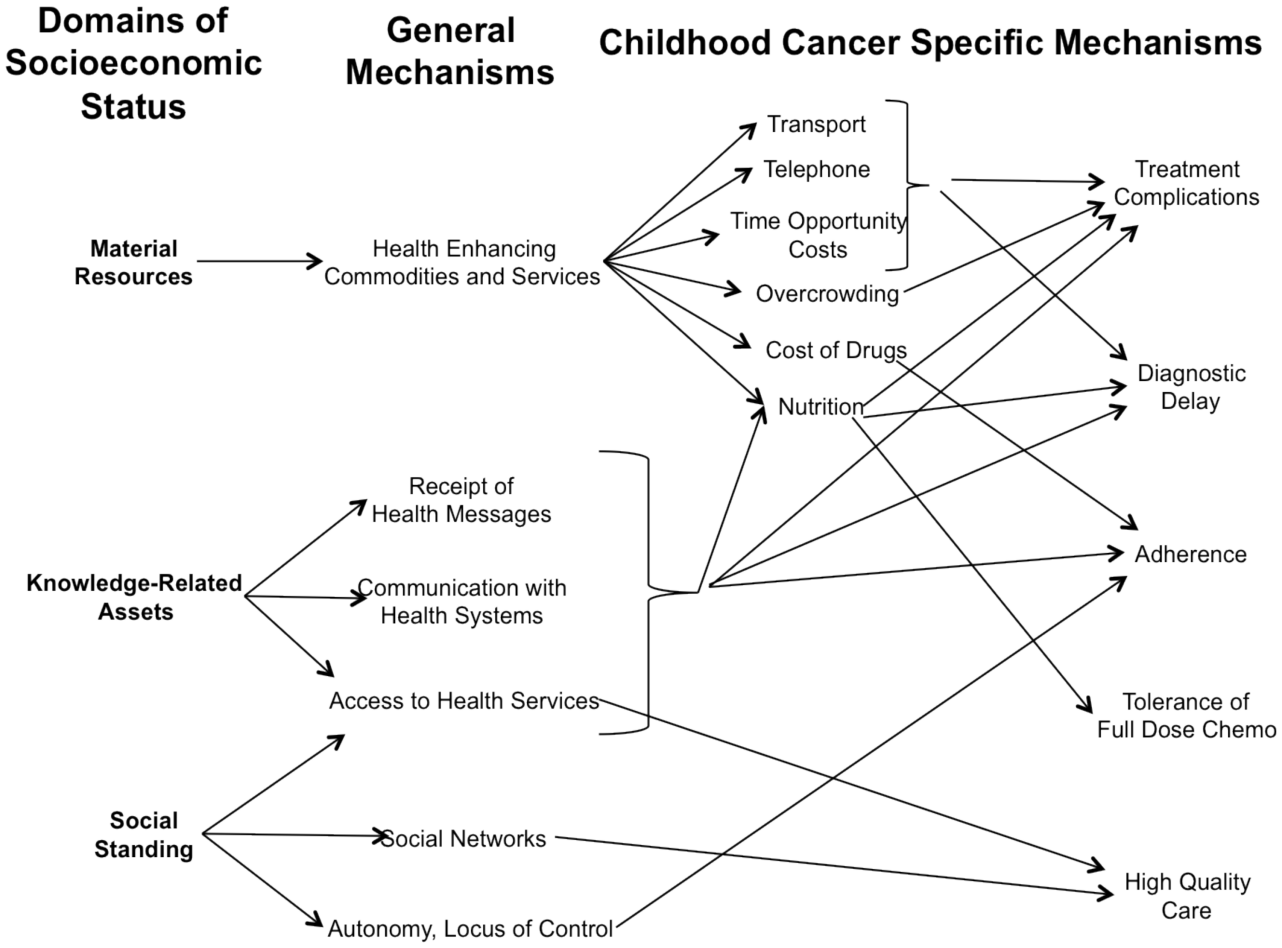
Mechanisms linking socioeconomic status domains to both general and childhood cancer specific health outcomes. Domains and general mechanisms are adapted from the work of Galobardes et?al., Braveman et?al., Krieger et?al. and Marmot. Several childhood specific mechanisms are suggested by Bhatia et?al., Gage, Viana et?al. and Gupta et?al. These mechanisms are often theoretical with little empiric basis.

We chose to include multiple measures of SES in this systematic review, as SES indicators measure “different, often related aspects of socioeconomic stratification and may be more or less relevant to different health outcomes.” [45] This issue may be particularly pronounced in pediatric oncology, where mechanisms linking SES and outcome are likely complex and inter-related, as illustrated in Figure 4. These mechanisms have been suggested by previous authors as outlined in the figure legend, but are often theoretical with little empiric basis.

Based on this framework, our finding that all measures of low SES in LMIC were associated with inferior outcome implies that in these settings, many mechanisms link SES and outcome. Interventions targeting a particular mechanism in LMIC are therefore likely to decrease but not erase socioeconomic gradients in outcome. For example, while the provision of free treatment, accommodation and transport to families in El Salvador resulted in a decrease in abandonment rates to 13%, socioeconomic variables remained the strongest predictors of abandonment. [46] Multi-faceted interventions are thus required in order to completely eliminate the negative influence of poor SES in LMIC.

Turning to studies conducted in HIC, income-based measures of SES were not significantly associated with outcome, though were infrequently investigated. By contrast, measures encompassing paternal education, material possession, and insurance status were often statistically associated with inferior outcome. This contrast to the LMIC findings has several potential explanations. First, a negative influence of low SES in HIC may be present but weaker than in LMIC, such that HIC studies were more likely to be underpowered. As the majority of non-significant associations were in the direction of low SES being associated with inferior outcome, this hypothesis is plausible.

Alternatively, only some of the pathways illustrated in Figure 4 may be relevant in HIC. Interestingly, both American studies examining the effect of insurance coverage found the lack of full coverage to be significantly associated with inferior survival. [34], [47] In HIC, measures of access to health care may therefore be more relevant than, for example, measures of income. It is also likely that the impact of different aspects of SES will vary between settings and malignancies. For example, different measures of SES are likely to be relevant in countries with universal access to health care than in those without. Compliance will have a greater potential effect upon outcome in malignancies for which outpatient oral chemotherapy plays a major role than those involving mainly inpatient therapy.

### Implications for Future Studies

Future studies must move beyond choosing socioeconomic variables and outcomes based simply on what data are easily available to the investigators. Instead, authors should posit specific mechanisms and potential confounders in advance, identify measures of SES and outcomes consistent with the hypothesis, and only then examine for significant associations. For example, Bhatia et?al. measured rates of compliance to oral chemotherapy among American children with ALL. Low rates of compliance were linked to single mother households and associated with higher rates of relapse. [48] Demonstrating the role of a particular pathway thus not only leads to a deeper understanding of the impact of SES, but also to plausible interventions mediating the pathway.

While such studies are likely to be complex, their impact may be significant. We have shown that improving the outcome of children of low SES to that of their high SES brethren would result in the elimination of up to 74.8% of adverse outcomes in LMIC and up to 31.9% of adverse outcomes in HIC. By way of comparison, minimal residual disease accounts for a theoretical 39.4% of relapse in high-risk ALL, while the novel feature of high CRLF2 expression accounts for 13.1% of relapse among all children with ALL. [49], [50] Consequently, debate on how low SES can be targeted is warranted, both in LMIC and HIC. Targeted interventions could encompass more frequent follow-up, intensive compliance monitoring, or other stratagems.

### Strengths and Limitations

This study represents the first comprehensive assessment of the effect of SES on children with cancer. Other strengths include the lack of language-based restrictions and the exclusion of ethnicity, allowing for the role of biologic confounders to be minimized. Our main limitation was the inability to compare magnitudes of associations across studies. Even when multiple studies used both the same outcome (e.g. EFS) and exposure (e.g. income), different units of analysis were used (richest income quintile vs. poorest income quintile, per $100 monthly income). In previous work we showed the effect of monthly income upon EFS in children with ALL in El Salvador was HR = 0.81 per $100. [28] Comparing the richest quartile to the poorest in the identical population would have resulted in a HR of 0.45. Thus meaningful comparisons can only be made when the analysis unit is identical. This also rendered the use of Forest plots inappropriate. Our figures instead were restricted to illustrating effect direction and significance. In the future, individual-level meta-analyses may be useful in this regard as long as the non-categorized covariate (e.g. monthly income) was collected. Secondly, it is possible that publication bias is present, particularly in studies of LMIC. Finally, the incidence of ALL has itself been linked to high SES in some studies. [51] For this to explain the findings of our systematic review, the biologic driver behind this association would have to be specific to a low-risk form of ALL across multiple populations. While we cannot rule this possibility out, this would not explain the association between SES and outcome seen in other cancers.

In conclusion, low SES is uniformly associated with poorer outcomes among LMIC children with cancer, and widespread among HIC children. Future studies should identify specific mechanisms underlying these gradients, as well as evaluate interventions aimed at improving the outcome of children with cancer with socioeconomic risk factors.

## Supporting Information

Figure S1 **Associations between socioeconomic measures and event-free and overall survival in studies conducted in the United States.** Positive = lower socioeconomic status associated with inferior outcome; Negative = lower socioeconomic status associated with superior outcome. Magnitudes of association are not plotted. Thus points distal from the y-axis may represent stronger, weaker or equivalent associations than proximal points.
(DOCX)

Table S1 **Eligible studies examining the impact of socioeconomic status upon outcome in children with cancer in low- and middle-income countries.** ALL – acute lymphoblastic leukemia; AML – acute myeloid leukemia; DFS – disease free survival; EFS – event free survival; HR – hazard ratio; N – number; OS – overall survival; SES – socioeconomic status; TRM – treatment related mortality. Bolded variables indicate statistically significant associations. ^a^The marginalization index used by Carlos et?al. is an ecologic measure of SES; all other variables in the table are measures of individual-level SES.
(DOCX)

Table S2 **Eligible studies examining the impact of socioeconomic status upon outcome in children with cancer in high-income countries.** ALL – acute lymphoblastic leukemia; AML – acute myeloid leukemia; CNS – central nervous system tumors; EFS – event free survival; ES – Ewing sarcoma; GCT – germ cell tumors; HR – hazard ratio; MB – medulloblastoma; N – number; NB – neuroblastoma; NHL – non-Hodgkin lymphoma; OR – odds ratio; OS – overall survival; OST – osteosarcoma; RR – relative risk; SES – socioeconomic status; STS – soft tissue sarcoma; UK – United Kingdom; USA – United States of America. Bolded variables indicate statistically significant associations. ^a^Individual malignancies within the overall category showed no significant association between SES and outcome. ^b^Adolescent and young adult population. ^c^Within the overall malignancy category, leukemias did show a significant association between lower SES and inferior outcome. ^d^Immigrant patients from one center were compared to a historical control. ^e^No statistical analysis was presented, though the authors state that survival was “directly related to SES”. ^f^HR is per level of occupation.
(DOCX)

Text S1 **Study Protocol.**
(DOCX)

Text S2 **Search Strategies.**
(DOCX)

Text S3 **Data Abstraction Form.**
(DOCX)

Checklist S1 **PRISMA Checklist.**
(DOC)
