# Supplementary material for: Low Socioeconomic Status Is Associated with Worse Survival in Children with Cancer: A Systematic Review
Source: PLoS One. 2014 Feb 26;9(2):e89482. doi: 10.1371/journal.pone.0089482 (PMC3935876; doi:10.1371/journal.pone.0089482)
Supplement: Figure S1 — Associations between socioeconomic measures and event-free and overall survival in studies conducted in the United States. Positive = lower socioeconomic status associated with inferior outcome; Negative = lower socioeconomic status associated with superior outcome. Magnitudes of association are not plotted. Thus points distal from the y-axis may represent stronger, weaker or equivalent associations than proximal points. (DOCX) [file pone.0089482.s001.docx]

**Figure S1. Associations between socioeconomic measures and event-free and overall survival in studies conducted in the United States.**


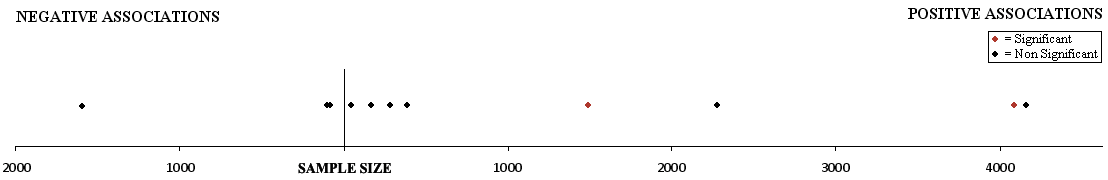


Positive = lower socioeconomic status associated with inferior outcome; Negative = lower socioeconomic status associated with superior outcome

Magnitudes of association are not plotted. Thus points distal from the y-axis may represent stronger, weaker or equivalent associations than proximal points
