# Supplementary material for: Low Socioeconomic Status Is Associated with Worse Survival in Children with Cancer: A Systematic Review
Source: PLoS One. 2014 Feb 26;9(2):e89482. doi: 10.1371/journal.pone.0089482 (PMC3935876; doi:10.1371/journal.pone.0089482)
Supplement: Table S1 — Eligible studies examining the impact of socioeconomic status upon outcome in children with cancer in low- and middle-income countries. ALL – acute lymphoblastic leukemia; AML – acute myeloid leukemia; DFS – disease free survival; EFS – event free survival; HR – hazard ratio; N – number; OS – overall survival; SES – socioeconomic status; TRM – treatment related mortality. Bolded variables indicate statistically significant associations. aThe marginalization index used by Carlos et?al. is an ecologic measure of SES; all other variables in the table are measures of individual-level SES. (DOCX) [file pone.0089482.s002.docx]

Table S1. Eligible studies examining the impact of socioeconomic status upon outcome in children with cancer in low- and middle-income countries

|  | Country | Malignancy | N | SES Variable | Definition | Subgroup | Outcome Measure | Outcome | Risk Measure |
| --- | --- | --- | --- | --- | --- | --- | --- | --- | --- |
| Bonilla 2010[^1^](#_ENREF_1) | El Salvador | Standard risk ALL | 260 | **Parental education** | **≥ Secondary vs. ≤ primary** | **.** | EFS | **.** | **HR 0.49 (0.29-0.84)** |
|  |  |  |  | Transport | Bus vs. own transport | . |  | . | HR 2.32 (0.57-9.45) |
|  |  |  |  | Telephone | Presence vs. absence | . |  | . | HR 0.61 (0.36-1.03) |
|  |  |  |  | **Monthly income** | **Per $100 increase** | **.** |  | **.** | **HR 0.84 (0.70-0.99)** |
|  |  | High risk ALL | 183 | Parental education | ≥ Secondary vs. ≤ primary | . | EFS | . | HR 0.69 (0.42-1.14) |
|  |  |  |  | Transport | Bus vs. own transport | . |  | . | HR 5.50 (0.76-39.78) |
|  |  |  |  | Telephone | Presence vs. absence | . |  | . | HR 0.70 (0.43-1.16) |
|  |  |  |  | Monthly income | Per $100 increase | . |  | . | HR 0.91 (0.80-1.03) |
| Mostert 2010[^2^](#_ENREF_2) | Indonesia | ALL | 283 | **Prosperity** | **2nd/3rd class ward vs VIP/1st class ward, based on income** | **.** | EFS | **.** | **HR 2.6 (1.8-3.7)** |
| Tang 2008[^3^](#_ENREF_3) | China | ALL | 346 | **Rurality/Insurance** | **.** | **Urban residence/ medical insurance** | 5 year EFS | **61.2%** | **Log rank p<0.0001** |
|  |  |  |  |  |  | **Rural residence/no medical insurance** |  | **30.3%** |  |
| Dinand 2007[^4^](#_ENREF_4) | India | Hodgkin Lymphoma | 145 | **Kuppuswami scale** | **Aggregate score based on income, education and occupation** | **High SES** | 5 year EFS | **93.2%** | **HR 5.4 (1.2-23.6)** |
|  |  |  |  |  |  | **Low SES** |  | **81.5%** |  |
| Pedrosa 2007[^5^](#_ENREF_5) | Brazil | Non-Hodgkin Lymphoma | 110 | Maternal education | . | Illiterate | OS | . | Log rank p=0.18 |
|  |  |  |  |  |  | ≤4th grade |  | . |  |
|  |  |  |  |  |  | >4th grade |  | . |  |
|  |  |  |  |  |  | Unknown |  | . |  |
|  |  |  |  | Family income | . | ≤2x minimum wage |  | . | Log rank p=0.21 |
|  |  |  |  |  |  | > 2x minimum wage |  | . |  |
|  |  |  |  |  |  | Unknown |  | . |  |
| Carlos 2002^a^[^6^](#_ENREF_6) | Mexico | Retinoblastoma | 552 | Marginalization index | **Multiple area-based social indicators** | **Very low (least marginalized)** | OS | **.** | **Reference** |
|  |  |  |  |  |  | **Low** |  | **.** | **HR 2.01 (1.14-3.55)** |
|  |  |  |  |  |  | **Medium** |  | **.** | **HR 2.26 (1.32-3.86)** |
|  |  |  |  |  |  | **High/very high (most marginalized)** |  | **.** | **HR 2.38 (1.40-4.04)** |
| Viana 1998[^7^](#_ENREF_7) | Brazil | ALL | 167 | **Monthly per capita income** |  | **<0.4 x minimum wage** | 5 year DFS | **58%** | **Log rank p<0.0001** |
|  |  |  |  |  |  | **>0.4 x minimum wage** |  | **8%** |  |
|  |  |  |  | **Mean daily electric energy consumption** |  | **>4kw hours** |  | **.** | **Log rank p=0.0003** |
|  |  |  |  |  |  | **<4kw hours** |  | **.** |  |
|  |  |  |  | **Housing conditions** | **Scale based on income, number of people, electric energy, physical characteristics** | **Fair-good** |  | **.** | **Log rank p=0.006** |
|  |  |  |  |  |  | **Intermediate** |  | **.** |  |
|  |  |  |  |  |  | **Very poor** |  | **.** |  |
| Gupta 2009[^8^](#_ENREF_8) | El Salvador | AML | 78 | Cost to travel to clinic | Per $1 increase | . | TRM | . | HR 1.08 (0.79-1.50) |
|  |  |  |  | Monthly income | Per $100 increase | . |  | . | HR 1.04 (0.89-1.22) |
|  |  |  |  | Telephone | Presence vs. absence | . |  | . | HR 1.04 (0.41-2.68) |
|  |  |  |  | Number of family members | Per family member | . |  | . | HR 0.98 (0.81-1.18) |
|  |  |  |  | Parental education | ≥ Secondary vs. ≤ primary | . |  | . | HR 0.94 (0.36-2.42) |
| Wang 2011[^9^](#_ENREF_9) | China | ALL | 323 | Paternal education |  | ≥High school | Abandonment | 62.2% | p=0.97 |
|  |  |  |  |  |  | ≤Junior school |  | 62.5% |  |
|  |  |  |  | Maternal education |  | ≥High school |  | 65.1% | p=0.47 |
|  |  |  |  |  |  | ≤Junior school |  | 60.1% |  |
|  |  |  |  | **Habitation condition** |  | **Good (≥2 of concrete, electricity, fridge)** |  | **32.5%** | **p<0.001** |
|  |  |  |  |  |  | **Poor** |  | **83.3%** |  |
| Kulkarni 2010[^10^](#_ENREF_10) | India | ALL | 532 | Kuppuswami scale | Aggregate score based on income, education and occupation | High SES | Abandonment | 12.4% | "Not significant" |
|  |  |  |  |  |  | Low SES |  | 22.5% |  |
|  |  |  |  |  |  |  |  |  |  |

ALL – acute lymphoblastic leukemia; AML – acute myeloid leukemia; DFS – disease free survival; EFS – event free survival; HR – hazard ratio; N – number; OS – overall survival; SES – socioeconomic status; TRM – treatment related mortality

Bolded variables indicate statistically significant associations

^a^The marginalization index used by Carlos et al. is an ecologic measure of SES; all other variables in the table are measures of individual-level SES

**REFERENCES**

**1.** Bonilla M, Gupta S, Vasquez R, et al. Predictors of outcome and methodological issues in children with acute lymphoblastic leukaemia in El Salvador. *Eur J Cancer.* Dec 2010;46(18):3280-3286.

**2.** Mostert S, Sitaresmi MN, Gundy CM, Janes V, Sutaryo, Veerman AJP. Comparing childhood leukaemia treatment before and after the introduction of a parental education programme in Indonesia. *Arch Dis Child.* Jan 2010;95(1):20-25.

**3.** Tang Y, Xu X, Song H, Yang S, Shi S, Wei J. Long-term outcome of childhood acute lymphoblastic leukemia treated in China. *Pediatr Blood Cancer.* Sep 2008;51(3):380-386.

**4.** Dinand V, Dawar R, Arya LS, Unni R, Mohanty B, Singh R. Hodgkin's lymphoma in Indian children: Prevalence and significance of Epstein-Barr virus detection in Hodgkin's and Reed-Sternberg cells. *Eur J Cancer.* Jan 2007;43(1):161-168.

**5.** Pedrosa MF, Pedrosa F, Lins MM, Pontes Neto NT, Falbo GH. Non-Hodgkin's lymphoma in childhood: clinical and epidemiological characteristics and survival analysis at a single center in Northeast Brazil. *J Pediatr (Rio J).* Nov-Dec 2007;83(6):547-554.

**6.** Carlos L-L, Roberto R-L, Victor T-G, Carlos H-G, Eduardo L-P. Risk of dying of retinoblastoma in Mexican children. *Med Pediatr Oncol.* Mar 2002;38(3):211-213.

**7.** Viana MB, Fernandes RA, de Carvalho RI, Murao M. Low socioeconomic status is a strong independent predictor of relapse in childhood acute lymphoblastic leukemia. *Int J Cancer Suppl.* 1998;11:56-61.

**8.** Gupta S, Bonilla M, Fuentes SL, et al. Incidence and predictors of treatment-related mortality in paediatric acute leukaemia in El Salvador. *Br J Cancer.* Apr 7 2009;100(7):1026-1031.

**9.** Wang YR, Jin RM, Xu JW, Zhang ZQ. A report about treatment refusal and abandonment in children with acute lymphoblastic leukemia in China, 1997-2007. *Leuk Res.* December 2011;35(12):1628-1631.

**10.** Kulkarni KP, Marwaha RK. Pattern and implications of therapy abandonment in childhood acute lymphoblastic leukemia. *Asian Pac J Cancer Prev.* 2010;11(5):1435-1436.
