# Supplementary material for: Low Socioeconomic Status Is Associated with Worse Survival in Children with Cancer: A Systematic Review
Source: PLoS One. 2014 Feb 26;9(2):e89482. doi: 10.1371/journal.pone.0089482 (PMC3935876; doi:10.1371/journal.pone.0089482)
Supplement: Table S2 — Eligible studies examining the impact of socioeconomic status upon outcome in children with cancer in high-income countries. ALL – acute lymphoblastic leukemia; AML – acute myeloid leukemia; CNS – central nervous system tumors; EFS – event free survival; ES – Ewing sarcoma; GCT – germ cell tumors; HR – hazard ratio; MB – medulloblastoma; N – number; NB – neuroblastoma; NHL – non-Hodgkin lymphoma; OR – odds ratio; OS – overall survival; OST – osteosarcoma; RR – relative risk; SES – socioeconomic status; STS – soft tissue sarcoma; UK – United Kingdom; USA – United States of America. Bolded variables indicate statistically significant associations. aIndividual malignancies within the overall category showed no significant association between SES and outcome. bAdolescent and young adult population. cWithin the overall malignancy category, leukemias did show a significant association between lower SES and inferior outcome. dImmigrant patients from one center were compared to a historical control. eNo statistical analysis was presented, though the authors state that survival was “directly related to SES”. fHR is per level of occupation. (DOCX) [file pone.0089482.s003.docx]

Supplemental Table 2. Eligible studies examining the impact of socioeconomic status upon outcome in children with cancer in high-income countries

|  | Country | Malignancy | N | SES Variable | Definition | Subgroup | Outcome Measure | Outcome | Risk Measure |
| --- | --- | --- | --- | --- | --- | --- | --- | --- | --- |
| Metzger 2008[^1^](#_ENREF_1) | USA | Hodgkin lymphoma | 327 | **%Children in poverty** | **Census-based county measure** | **<23%** | **EFS** | **.** | **Reference** |
|  |  |  |  |  |  | **>23.1%** |  | **.** | **1.9 (1.1-3.2)** |
| Bhatia 2002[^2^](#_ENREF_2) | USA, Canada | ALL | 1596 | Maternal education | >High school | . | EFS | . | Reference |
|  |  |  |  |  | ≤High school | . |  | . | HR 0.99 (0.8-1.2) |
|  |  |  |  | Paternal education | >High school | . |  | . | Reference |
|  |  |  |  |  | ≤High school | . |  | . | HR 1.2 (0.9-1.5) |
|  |  |  |  | Annual household income | >$30,000 | . |  | . | Reference |
|  |  |  |  |  | ≤$30,000 | . |  | . | HR 1.0 (0.8-1.3) |
| Hann 1981[^3^](#_ENREF_3) | England | ALL | 209 | Paternal occupation | . | Profession/managerial (I) | 5 year EFS | 18% | Log rank p=0.11 |
|  |  |  |  |  |  | Clerical (II) |  | 27% |  |
|  |  |  |  |  |  | Skilled (III) |  | 26% |  |
|  |  |  |  |  |  | Semi-skilled (IV) |  | 33% |  |
|  |  |  |  |  |  | Unskilled (V) |  | 42% |  |
| Lightfoot 2012[^4^](#_ENREF_4) | England, Scotland, Wales | ALL | 1559 | **Deprivation score** | **Census-based multi-item area deprivation index** | **Q1-Q3 (Affluent)** | **10 year OS** | **.** | **Reference** |
|  |  |  |  |  |  | **Q4-Q5 (Deprived)** |  | **.** | **HR 1.29 (1.05-1.57)** |
|  |  |  |  | Paternal Occupation |  | Professional/managerial |  | . |  |
|  |  |  |  |  |  | Skilled manual/non-manual |  | . |  |
|  |  |  |  |  |  | Semi-skilled/unskilled |  | . | HR 1.12 (0.97-1.29)^f^ |
| Syse 2012[^5^](#_ENREF_5) | Norway | Cancers | 6280 | **Maternal education** | **.** | **≥College** | **OS** | **.** | **Reference** |
|  |  |  |  |  |  | **≤High school** |  | **.** | **OR 1.2 (1.04-1.39)** |
|  |  |  |  | Marital status | . | Married |  | . | Reference |
|  |  |  |  |  |  | Unmarried |  | . | OR 0.99 (0.87-1.11) |
|  |  |  |  | Household income | . | <$10,000 |  | . | OR 1.09 (0.88-1.35) |
|  |  |  |  |  |  | $10,000-$19,999 |  | . | OR 1.00 (0.81-1.23) |
|  |  |  |  |  |  | $20,000-$39,999 |  | . | OR 0.95 (0.79-1.13) |
|  |  |  |  |  |  | $40,000-$59,999 |  | . | Reference |
|  |  |  |  |  |  | $60,000-$79,999 |  | . | OR 0.97 (0.78-1.20) |
|  |  |  |  |  |  | $80,000-$99,999 |  | . | OR 0.91 (0.69-1.19) |
|  |  |  |  |  |  | ≥$100,000 |  | . | OR 0.96 (0.73-1.26) |
|  |  |  |  | Number of children | . | 0 |  | . | OR 0.82 (0.69-0.97) |
|  |  |  |  |  |  | 1 |  | . | Reference |
|  |  |  |  |  |  | ≥2 |  | . | OR 0.96 (0.86-1.07) |
| Rondelli 2011[^6^](#_ENREF_6) | Italy | ALL | 3522 | **Immigrant status** | **.** | **Non-immigrant** | **5 year OS** | **86.6%** | **Reference** |
|  |  |  |  |  |  | **Immigrant** |  | **71.0%** | **HR 1.70 (1.16-2.50)** |
| Walsh 2011^a^ [^7^](#_ENREF_7) | Ireland | All Cancers | 1440 | SAHRU Deprivation Index | Census-based multi-item area deprivation index | 1 to 2 (least deprived) | 5 year OS | 79% |  |
|  |  |  |  |  |  | 3 to 4 |  | 83% |  |
|  |  |  |  |  |  | 5 to 6 |  | 77% |  |
|  |  |  |  |  |  | 7 to 8 |  | 75% |  |
|  |  |  |  |  |  | 9 to 10 (most deprived) |  | 80% |  |
| Youlden 2011[^8^](#_ENREF_8) | Australia | Cancers | 6289 | **Remoteness** | **Based on distance to closest service centers** | **Major city** | **5 year OS** | **81.6%** | **Reference** |
|  |  |  |  |  |  | **Inner regional** |  | **79.6%** | **HR 1.12 (0.94-1.34)** |
|  |  |  |  |  |  | **Outer regional** |  | **79.2%** | **HR 1.15 (0.92-1.29)** |
|  |  |  |  |  |  | **Remote/very remote** |  | **73.3%** | **HR 1.55 (1.08-2.23)** |
|  |  |  |  | Index of Relative Socioeconomic Disadvantage | Census-based multi-item area deprivation index | Least disadvantaged (Q5) |  | 82.6% | HR 0.86 (0.71-1.05) |
|  |  |  |  |  |  | Middle SES (Q2-Q4) |  | 80.3% | Reference |
|  |  |  |  |  |  | Most disadvantaged (Q1) |  | 78.5% | HR 1.10 (0.90-1.36) |
| Crouch 2009^b^ [^9^](#_ENREF_9) | UK | All cancers | 654 | **Multiple Deprivation Index** | **Census-based multi-item area deprivation index** | **Q1 (affluent)** | **5 year OS** | **70%** | **Test for trend p<0.05** |
|  |  |  |  |  |  | **Q2** |  | **71%** |  |
|  |  |  |  |  |  | **Q3** |  | **69%** |  |
|  |  |  |  |  |  | **Q4** |  | **66%** |  |
|  |  |  |  |  |  | **Q5 (deprived)** |  | **64%** |  |
| Hsieh 2009[^10^](#_ENREF_10) | USA | NB | 1777 | **Rurality** | **SEER-based study** | **Metropolitan county** | **5 year OS** | **63%** | **Log rank p=0.04** |
|  |  |  |  |  |  | **Non-metropolitan county** |  | **55%** |  |
| Kent 2009[^11^](#_ENREF_11) | USA | Leukemias | 4158 | SES | Census-based multi-item area deprivation index | Highest SES | OS | . | Reference |
|  |  |  |  |  |  | High SES |  | . | HR 1.15 (0.88-1.50) |
|  |  |  |  |  |  | Middle SES |  | . | HR 0.99 (0.75-1.30) |
|  |  |  |  |  |  | Low SES |  | . | HR 1.09 (0.84-1.43) |
|  |  |  |  |  |  | Lowest SES |  | . | HR 1.13 (0.86-1.47) |
|  |  |  |  | **Insurance** | **.** | **Any** |  | **.** | **Reference** |
|  |  |  |  |  |  | **None/unknown** |  | **.** | **HR 1.56 (1.26-1.94)** |
| Birch 2008^a,b^ [^12^](#_ENREF_12) | England | All Cancers | 31722 | **Townsend Deprivation Index** | **Census-based multi-item area deprivation index** | **Q1 (affluent)** | **5 year OS** | **71%** | **Test for trend p=0.001** |
|  |  |  |  |  |  | **Q2** |  | **71%** |  |
|  |  |  |  |  |  | **Q3** |  | **72%** |  |
|  |  |  |  |  |  | **Q4** |  | **71%** |  |
|  |  |  |  |  |  | **Q5 (deprived)** |  | **70%** |  |
| Moschovi 2007[^13^](#_ENREF_13) | Greece | MB | 50 | Maternal Education | "Per level" | . | OS | . | HR 1.33 (0.74-2.4) |
|  |  |  |  | Place of residence | "Proxy for access" | Urban |  | . | Reference |
|  |  |  |  |  |  | Rural |  | . | HR 3.43 (0.91-13.0) |
| Perez-Martinez 2007^d^ [^14^](#_ENREF_14) | Spain | All cancers | 90+ | Immigrant status | . | Non-immigrant | 5 year OS | 73% | . |
|  |  |  |  |  |  | Immigrant |  | 61% | . |
| Tseng 2006[^15^](#_ENREF_15) | England, Wales | Malignant CNS | 3169 | Carstairs index | Census-based multi-item area deprivation index | Q1 (most affluent) | 5 year OS | 53.8% | Log rank p=0.54 |
|  |  |  |  |  |  | Q2 |  | 55.2% |  |
|  |  |  |  |  |  | Q3 |  | 48.8% |  |
|  |  |  |  |  |  | Q4 |  | 49.2% |  |
|  |  |  |  |  |  | Q5 (least affluent) |  | 53.3% |  |
| Charalampopolou 2004[^16^](#_ENREF_16) | Greece | ALL | 293 | Maternal education | <10 years vs. ≥10 years | . | OS | . | HR 1.67 (0.85-3.31) |
|  |  |  |  | **Marital status** | **Other vs. married** | **.** |  | **.** | **HR 2.85 (1.19-6.81)** |
|  |  |  |  | **N of children** | **Per child** | **.** |  | **.** | **HR 0.63 (0.40-0.99)** |
| Coleman 1999[^17^](#_ENREF_17) | England, Wales | Hodgkin lymphoma | 189 | Castairs Index | Census-based multi-item area deprivation index | Affluent | 5 year OS | 95.0% |  |
|  |  |  |  |  | Difference in survival between affluent and deprived group (negative indicates lower survival in deprived quintile) | Gap |  | -3.9 (-13.4 to 5.6) |  |
|  |  | NHL | 273 |  |  | Affluent |  | 80.9% |  |
|  |  |  |  |  |  | Gap |  | -9.0 (-23.4 to 5.5) |  |
|  |  | CNS | 1050 |  |  | Affluent |  | 61.9% |  |
|  |  |  |  |  |  | Gap |  | -6.0 (-14.4 to 2.3) |  |
|  |  | Wilms | 257 |  |  | Affluent |  | 83.0% |  |
|  |  |  |  |  |  | Gap |  | 4.2 (-9.7 to 18.1) |  |
|  |  | OST | 117 |  |  | Affluent |  | 57.3% |  |
|  |  |  |  |  |  | Gap |  | -15.6 (-40.0 to 8.8) |  |
|  |  | ES | 97 |  |  | Affluent |  | 60.9% |  |
|  |  |  |  |  |  | Gap |  | -10.1 (-38.3 to 18.2) |  |
|  |  | STS | 319 |  |  | Affluent |  | 62.3% |  |
|  |  |  |  |  |  | Gap |  | 8.0 (-7.3 to 23.3) |  |
|  |  | GCT | 121 |  |  | Affluent |  | 78.5% |  |
|  |  |  |  |  |  | Gap |  | 12.4 (-3.2 to 28.0) |  |
| McKinney 1999^c^ [^18^](#_ENREF_18) | UK | All Cancers | 1979 | Carstairs index | Census-based multi-item area deprivation index | Q1 (affluent) | OS | 57.7% | Log rank p=0.05 |
|  |  |  |  |  |  | Q2 |  | 61.4% |  |
|  |  |  |  |  |  | Q3 |  | 56.6% |  |
|  |  |  |  |  |  | Q4 |  | 54.8% |  |
|  |  |  |  |  |  | Q5 (deprived) |  | 52.0% |  |
| Schillinger 1999[^19^](#_ENREF_19) | England, Wales | ALL | 5566 | Castairs Index | Census-based multi-item area deprivation index | Q1 (affluent) | 5 year OS | 79% | Log rank p=0.77 |
|  |  |  |  |  |  | Q2 |  | 76% |  |
|  |  |  |  |  |  | Q3 |  | 78% |  |
|  |  |  |  |  |  | Q4 |  | 74% |  |
|  |  |  |  |  |  | Q5 (deprived) |  | 76% |  |
| Coebergh 1996[^20^](#_ENREF_20) | Netherlands | Standard-risk ALL | 367 | Parental education | Higher | . | 5 year OS | 70% | p=0.25 |
|  |  |  |  |  | Primary/low vocational | . |  | 60% |  |
|  |  | High-risk ALL | 141 | Parental education | Higher | . |  | 34% | p=0.10 |
|  |  |  |  |  | Primary/low vocational | . |  | 48% |  |
|  |  | AML | 67 | Parental education | Higher | . |  | 12% | p=0.22 |
|  |  |  |  |  | Primary/low vocational | . |  | 19% |  |
| Hord 1996[^21^](#_ENREF_21) | USA | ALL | 178 | **Pay status** | **Total coverage vs. at least partially uncovered** | **.** | **5 year OS** | **.** | **OR 0.61 (0.27-0.79)** |
| Petridou 1994[^22^](#_ENREF_22) | Greece | Leukemias | 120 | Maternity hospital | . | Public | OS | . | Reference |
|  |  |  |  |  |  | Private |  | . | HR 0.57 (0.26-1.22) |
|  |  |  |  | Paternal occupation | . | Manual |  | . | Reference |
|  |  |  |  |  |  | Nonmanual |  | . | HR 0.75 (0.34-1.64) |
|  |  |  |  | Paternal education | . | ≤11 years |  | . | Reference |
|  |  |  |  |  |  | >11 years |  | . | HR 0.68 (0.31-1.46) |
|  |  |  |  | Maternal education | . | ≤11 years |  | . | Reference |
|  |  |  |  |  |  | >11 years |  | . | HR 0.87 (0.20-1.87) |
|  |  |  |  | **Private car** | **.** | **No** |  | **.** | **Reference** |
|  |  |  |  |  |  | **Yes** |  | **.** | **HR 0.29 (0.13-0.62)** |
|  |  |  |  | Free choice doctor | . | No |  | . | Reference |
|  |  |  |  |  |  | Yes |  | . | HR 0.96 (0.41-2.23) |
| McWhirter 1983[^23^](#_ENREF_23) | Australia | ALL | 70 | **Parental education** | **Occupations divided into social classes** | **Higher SES** | **5 year OS** | **59%** | **p=0.03** |
|  |  |  |  |  |  | **Lower SES** |  | **27%** |  |
| Szklo 1978[^24^](#_ENREF_24) | USA | ALL | 55 | **Median rental value** | **Census-based** | **High (Q4 and Q5)** | **2 year OS** | **51%** | **p<0.005** |
|  |  |  |  |  |  | **Low (Q1-Q3)** |  | **28%** |  |
| Byrne 2011[^25^](#_ENREF_25) | USA | AML  (Age 0-9) | 84 | Community poverty level | Census-based | <5% | Median duration | 11.2 mo |  |
|  |  |  |  |  |  | 5.1-10% |  | 19.7 mo |  |
|  |  |  |  |  |  | 10.1-15% |  | n/a |  |
|  |  |  |  |  |  | >15% |  | 21.8 mo |  |
|  |  | AML  (Age 10-19) | 102 | Community poverty level | Census-based | <5% |  | 7.2 mo |  |
|  |  |  |  |  |  | 5.1-10% |  | 10.4 mo |  |
|  |  |  |  |  |  | 10.1-15% |  | 16.4 mo |  |
|  |  |  |  |  |  | >15% |  | 9.2 mo |  |
| Walters 1972^e^ [^26^](#_ENREF_26) | USA | ALL | 334 | **Paternal occupation** | **SES groups based on occupation** | **Group 1 (lowest SES)** | **Median duration** | **16.2 mo** |  |
|  |  |  |  |  |  | **Group 2** |  | **21.8 mo** |  |
|  |  |  |  |  |  | **Group 3** |  | **26.8 mo** |  |
|  |  |  |  |  |  | **Group 4 (highest SES)** |  | **24.3 mo** |  |
|  |  |  |  |  |  |  |  |  |  |

ALL – acute lymphoblastic leukemia; AML – acute myeloid leukemia; CNS – central nervous system tumors; EFS – event free survival; ES – Ewing sarcoma; GCT – germ cell tumors; HR – hazard ratio; MB – medulloblastoma; N – number; NB – neuroblastoma; NHL – non-Hodgkin lymphoma; OR – odds ratio; OS – overall survival; OST – osteosarcoma; RR – relative risk; SES – socioeconomic status; STS – soft tissue sarcoma; UK – United Kingdom; USA – United States of America

Bolded variables indicate statistically significant associations

^a^Individual malignancies within the overall category showed no significant association between SES and outcome

^b^Adolescent and young adult population

^c^Within the overall malignancy category, leukemias did show a significant association between lower SES and inferior outcome

^d^Immigrant patients from one center were compared to a historical control

^e^No statistical analysis was presented, though the authors state that survival was “directly related to SES”

^f^HR is per level of occupation

**REFERENCES**

**1.** Metzger ML, Castellino SM, Hudson MM, et al. Effect of race on the outcome of pediatric patients with Hodgkin's lymphoma. *J Clin Oncol.* Mar 10 2008;26(8):1282-1288.

**2.** Bhatia S, Sather HN, Heerema NA, Trigg ME, Gaynon PS, Robison LL. Racial and ethnic differences in survival of children with acute lymphoblastic leukemia. *Blood.* Sep 15 2002;100(6):1957-1964.

**3.** Hann IM, Palmer MK, Morris-Jones PH, Evans DI. Childhood leukaemia and social status. *Lancet.* Dec 5 1981;2(8258):1282-1283.

**4.** Lightfoot TJ, Johnston WT, Simpson J, et al. Survival from childhood acute lymphoblastic leukaemia: the impact of social inequality in the United Kingdom. *Eur J Cancer.* 2012;48(2):263-269.

**5.** Syse A, Lyngstad TH, Kravdal O. Is mortality after childhood cancer dependent on social or economic resources of parents? A population-based study. *Int J Cancer.* 1870;130(8):1870-1878.

**6.** Rondelli R, Dini G, De Rosa M, et al. Foreign children with cancer in Italy. *Italian Journal of Pediatrics.* 2011;37(1).

**7.** Walsh PM, Byrne J, Capra M, Comber H. Childhood cancer survival in Ireland: temporal, regional and deprivation-related patterns. *Eur J Cancer.* Aug 2011;47(12):1852-1862.

**8.** Youlden DR, Baade PD, Valery PC, Ward LJ, Green AC, Aitken JF. Differentials in survival for childhood cancer in Australia by remoteness of residence and area disadvantage. *Cancer Epidemiol Biomarkers Prev.* 1649;20(8):1649-1656.

**9.** Croucher C, Whelan JS, Moller H, Davies EA. Trends in the incidence and survival of cancer in teenagers and young adults: regional analysis for South East England 1960-2002. *Clin Oncol (R Coll Radiol).* Jun 2009;21(5):417-424.

**10.** Hsieh MH, Meng MV, Walsh TJ, Matthay KK, Baskin LS. Increasing incidence of neuroblastoma and potentially higher associated mortality of children from nonmetropolitan areas: analysis of the surveillance, epidemiology, and end results database. *J Pediatr Hematol Oncol.* Dec 2009;31(12):942-946.

**11.** Kent EE, Sender LS, Largent JA, Anton-Culver H. Leukemia survival in children, adolescents, and young adults: influence of socioeconomic status and other demographic factors. *Cancer Causes Control.* Oct 2009;20(8):1409-1420.

**12.** Birch JM, Pang D, Alston RD, et al. Survival from cancer in teenagers and young adults in England, 1979-2003. *Br J Cancer.* Sep 2 2008;99(5):830-835.

**13.** Moschovi M, Stavrou T, Dessypris N, et al. Survival among children with medulloblastoma in Greece: gains from transition to chemotherapy and socio-economic differentials. *Eur J Cancer Prev.* Oct 2007;16(5):460-465.

**14.** Perez-Martinez A, del Palacio EC, Nunez-Polo MH, Olivares JAB, Orellana MR, Lopez LM. Childhood cancer in immigration population needs "AMOR" too. *Pediatr Blood Cancer.* Nov 2007;49(6):876-877.

**15.** Tseng JH, Tseng MY. Survival analysis of children with primary malignant brain tumors in England and Wales: A population-based study. *Pediatr Neurosurg.* Feb 2006;42(2):67-73.

**16.** Charalampopoulou A, Petridou E, Spyridopoulos T, et al. An integrated evaluation of socioeconomic and clinical factors in the survival from childhood acute lymphoblastic leukaemia: a study in Greece. *Eur J Cancer Prev.* Oct 2004;13(5):397-401.

**17.** Coleman MP. Cancer survival trends in England and Wales, 1971-1995: deprivation and NHS region. London: The Stationery Office; 1999.

**18.** McKinney PA, Feltbower RG, Parslow RC, et al. Survival from childhood cancer in Yorkshire, U.K.: effect of ethnicity and socio-economic status. *Eur J Cancer.* Dec 1999;35(13):1816-1823.

**19.** Schillinger JA, Grosclaude PC, Honjo S, Quinn MJ, Sloggett A, Coleman MP. Survival after acute lymphocytic leukaemia: effects of socioeconomic status and geographic region. *Arch Dis Child.* Apr 1999;80(4):311-317.

**20.** Coebergh JW, van der Does-van den Berg A, Hop WC, et al. Small influence of parental educational level on the survival of children with leukaemia in The Netherlands between 1973 and 1979. *Eur J Cancer.* Feb 1996;32A(2):286-289.

**21.** Hord MH, Smith TL, Culbert SJ, Frankel LS, Pinkel DP. Ethnicity and cure rates of Texas children with acute lymphoid leukemia. *Cancer.* 01 Feb 1996;77(3):563-569.

**22.** Petridou E, Kosmidis H, Haidas S, et al. Survival from childhood leukemia depending on socioeconomic status in Athens. *Oncology.* Sep-Oct 1994;51(5):391-395.

**23.** McWhirter WR, Smith H, McWhirter KM. Social class as a prognostic variable in acute lymphoblastic leukaemia. *Med J Aust.* Oct 1 1983;2(7):319-321.

**24.** Szklo M, Gordis L, Tonascia J, Kaplan E. The changing survivorship of white and black children with leukemia. *Cancer.* Jul 1978;42(1):59-66.

**25.** Byrne MM, Halman LJ, Koniaris LG, Cassileth PA, Rosenblatt JD, Cheung MC. Effects of poverty and race on outcomes in acute myeloid leukemia. *Am J Clin Oncol.* Jun 2011;34(3):297-304.

**26.** Walters TR, Bushore M, Simone J. Poor prognosis in Negro children with acute lymphocytic leukemia. *Cancer.* Jan 1972;29(1):210-214.
