# Supplementary material for: Low Socioeconomic Status Is Associated with Worse Survival in Children with Cancer: A Systematic Review
Source: PLoS One. 2014 Feb 26;9(2):e89482. doi: 10.1371/journal.pone.0089482 (PMC3935876; doi:10.1371/journal.pone.0089482)
Supplement: Text S1 — Study Protocol. (DOCX) [file pone.0089482.s004.docx]

**The Association between Socioeconomic Status and Outcome Among Children with Cancer: A Systematic Review**

Sumit Gupta, Astrid Guttmann, Jason Pole, Lillian Sung

Working Protocol v.3

December 15th, 2011

**RATIONALE**

Efforts to ascertain the underlying cause of treatment failure among children with cancer have predominantly focused on biologic factors such as cancer cytogenetics and host polymorphisms^1, 2^. The potential explanatory role of socioeconomic status (SES) has been relatively ignored. Indeed, most studies examining the association between SES and cancer outcomes have been conducted in adult populations. A recent review concluded that strong evidence exists for an association between lower SES and higher adult cancer mortality across different measures of SES^3^. These associations have also been demonstrated in Ontario^4^, despite universal access healthcare.

By contrast, the equivalent paediatric literature is sparse and predominantly restricted to low income countries. In that context, various measures of SES have predicted outcome in both settings where the cost of treatment was borne by families and where the provision of treatment was free.^5-7^ Among children with ALL in El Salvador for example, lower parental income and/or education predicted the risk of specific causes of treatment failure (e.g. treatment-related mortality, abandonment of therapy) as well as worse overall survival^8-10^. Interestingly, we demonstrated a negative effect of lower SES on survival among children with standard-risk ALL but not in high-risk ALL, suggesting that in high-risk children, a combination of aggressive disease and high intensity treatment may overwhelm any protective mechanisms of high SES^10^. Various mechanisms underlying this association have been suggested, including an inability to absorb opportunity costs, poor understanding of the disease, differential attitudes and practices of health care providers and poor compliance.^6, 8, 11, 12^ The generalizability of these findings to high income countries is, however, highly questionable.

Those few studies in high income countries have yielded contradictory results. One of the first was conducted in Australia and noted an association between low area-level social class (based on various measures including education and occupation) and inferior outcome in ALL, finding the strongest association in low risk children (those with presenting WBC counts < 30 x10^9^/L)^13^. Other studies in Europe found single motherhood and low education to be associated with inferior survival, though a population-based study in the United Kingdom found only survival gradients based on region, and not based on area-level material deprivation^14, 15^. In the United States, most research has focused on racial differences in survival, recognizing that ethnicity is “closely linked with SES”^16^. In a retrospective study of 8,447 children enrolled on Children’s Oncology Group therapeutic ALL protocols, Bhatia et al. found race, but not parental income or education, to predict survival^17^. The authors however noted that SES data was available for only a small proportion of the study population, with consequent concerns over generalizability and low power. Indeed, further doubt is raised by the fact that families of low SES who choose to participate in clinical trials may be systematically different to those who refuse participation^18, 19^. Equivalent studies in the Canadian context are lacking.

Thus, whether SES gradients in paediatric cancer outcome exist in high income countries with universal health care access is unknown. A systematic review of studies describing the association between measures of SES and outcomes in children with cancer is therefore warranted in order to synthesize the current state of knowledge, highlight gaps in the literature and identify future avenues of investigation.

This systematic review will follow the guidelines for reporting systematic reviews as outlined by the PRISMA statement and will be registered on Prospero^20^.

**OBJECTIVES**

Primary

1. To determine association between SES and event free survival among paediatric oncology patients

Secondary

1. To determine association between SES and other outcomes, including overall survival, relapse, disease free survival, abandonment of therapy and treatment related mortality, among paediatric oncology patients

Exploratory

1. To determine the effect of patient level variables (e.g. malignancy type) on the association between SES and cancer outcome
2. To determine the effect of centre level variables (e.g. high income country vs. low/middle income country) on the association between SES and cancer outcome

**METHODS**

Eligibility Criteria

Inclusion Criteria

1. Ecologic, cross-sectional, cohort, case-control or randomized control trial design
2. Pediatric oncology patients, with age defined as the study with pediatric data abstractable
3. At least one primary survival-related outcome (eg. event free survival (EFS), overall survival (OS), disease free survival (DFS), treatment related mortality (TRM), relapse, abandonment of therapy)
4. Outcome reported by subgroups defined by a socioeconomic variable, including both ecologic and individual level measures of income, education, material possession, family composition, insurance status, health care accessibility or immigrant status

Exclusion Criteria

1. Duplicate reporting – same group of patients
2. Adult population (as defined by authors) or no pediatric data abstractable
3. Non-malignant diagnosis
4. No survival-related outcome (EFS, OS, DFS, TRM, relapse, abandonment of therapy)
5. No subgroups defined by a socioeconomic variable (including both ecologic and individual level measures of income, education, material possession, family composition, insurance status, health care accessibility or immigrant status) with outcome reported. NB that race/ethnicity is not a valid socioeconomic variable in this systematic review
6. Only population-based outcome available (e.g. mortality per 100,000 children)
7. Narrative or systematic review without any original data
8. Not eligible study design (ecologic, cross-sectional, cohort, case-control or randomized control trial)

Information Sources

An electronic search of OVID MEDLINE, CINAHL and EMBASE will be conducted from database inception to the present day in order to identify studies.

Search

Using previously validated filters for studies of prognosis, search terms for various measures of SES will be used and will be restricted to paediatric oncology patients. Examples of search terms that will be used to identify studies include (but are not limited to):

Socio-economic status

Social class

Poverty

Educational status

Deprivation

Disparities

Regional Variation

Insurance

Studies will not be restricted by language. It is expected that the search will be iterative in nature; new search terms will be identified when reviewing studies and will be included in the search strategy. Consultation with a librarian scientist will occur throughout this process.

Study Selection

After the identification and deletion of duplicate publications, two individuals will independently screen whether the remaining abstracts potentially meet the eligibility criteria. Each record will be independently screened by 2 reviewers. Any article potentially eligible as identified by either reviewer will be retrieved in full. The full articles will then be evaluated for eligibility. Inclusion in the systematic review will be by consensus of both reviewers. In the case of disagreement, a third reviewer will arbitrate if the two reviewers cannot come to consensus. Agreement on inclusion of retrieved articles into the final review will be evaluated by the kappa statistic.

Data Collection

Data of interest will be collected by one of several investigators and recorded on a pre-determined data extraction form (see separate document). In instances in which data fields of interest are not available from the text of the manuscript, authors will be contacted to try to obtain missing data. Should a response not be received within two weeks of the original attempt at contact, a second attempt will be made. No further contacts will be initiated.

Data Items

Data elements to be abstracted from included studies can be categorized under several headings:

Characteristics of underlying population – number of patients, malignancy type, age range, geographic location, single vs. multi-centre

Treatment variables - treatment protocol, chemotherapy, surgery, radiation, transplant

Outcome – specific measure of outcome, outcome for overall group

SES variable - specific measure of SES, individual vs. ecologic level, quantitative strength of association between SES variable and outcome

Study quality indicators

An initial version of the data extraction form is shown in Appendix 1. This form will be piloted with initially identified studies and then modified as needed.

Risk of Bias in Individual Studies

The PRISMA statement advises that assessing bias required both “study-level” assessment and “outcome-level” assessment. Both are accounted for in the framework of potential biases outlined by Hayden et al^22^. This framework will therefore be used to assess study quality. The specific variables to be abstracted are also presented in the Appendix.

Summary Measures, Synthesis of Results and Additional Analyses

Given the different measures of SES used and the possible heterogeneous effect of SES depending on malignancy type or geography, quantitatively pooling studies is not likely to be informative. Thus, we will conduct a qualitative systematic review and will stratify results by studies from high or low income countries, as per the World Bank definition based on 2010 Gross National Income per capita. Should sufficient studies be identified, a further stratification by malignancy type will be undertaken.

Risk of Bias Across Studies

It is possible that some prospective treatment trials have collected information on SES as a secondary aim and, while reporting outcome data based on SES in the text, do not do so in the title, abstract or keywords. Our search strategy may miss such studies. As negative associations are more likely to be reported in the text and not the title, abstract or keywords, bias may be introduced into our summary. We do not anticipate this to be a major problem as anecdotal evidence suggests that collecting SES information on trials is not commonly done. Indeed an expert has recently highlighted this lack^23^. In order to formally assess the magnitude of this problem however, we will identify all prospective treatment trials conducted by the major cooperative trial groups in ALL, the most common malignancy, over the last 15 years. The principle investigators of each trial will be contacted and asked whether any information on SES was collected, and if so, whether any analysis using this information was conducted.

REFERENCES

1. Mullighan CG, Downing JR. Genome-wide profiling of genetic alterations in acute lymphoblastic leukemia: Recent insights and future directions. Leukemia 2009;23:1209-18.

2. Schmiegelow K, Forestier E, Kristinsson J, et al. Thiopurine methyltransferase activity is related to the risk of relapse of childhood acute lymphoblastic leukemia: results from the NOPHO ALL-92 study. Leukemia 2009;23:557-64.

3. Woods LM, Rachet B, Coleman MP. Origins of socio-economic inequalities in cancer survival: a review. Annals of Oncology 2006;17:5-19.

4. Booth CM, Li G, Zhang-Salomons J, Mackillop WJ. The impact of socioeconomic status on stage of cancer at diagnosis and survival. Cancer 2010;116:4160-7.

5. Tang Y, Xu X, Song H, Yang S, Shi S, Wei J. Long-term outcome of childhood acute lymphoblastic leukemia treated in China. Pediatr Blood Cancer 2008;51:380-6.

6. Mostert S, Sitaresmi MN, Gundy CM, Sutaryo, Veerman AJ. Influence of socioeconomic status on childhood acute lymphoblastic leukemia treatment in Indonesia. Pediatrics 2006;118:e1600-6.

7. Viana MB, Fernandes RA, de Carvalho RI, Murao M. Low socioeconomic status is a strong independent predictor of relapse in childhood acute lymphoblastic leukaemia. Int J Cancer (Suppl) 1998;11:56-61.

8. Gupta S, Bonilla M, Fuentes SL, et al. Incidence and predictors of treatment-related mortality in paediatric acute leukaemia in El Salvador. Br J Cancer 2009;100:1026-31.

9. Bonilla M, Rossell N, Salaverria C, et al. Prevalence and predictors of abandonment of therapy among children with cancer in El Salvador. Int J Cancer 2009;125:2144-6.

10. Bonilla M, Gupta S, Vasquez R, et al. Predictors of outcome and methodological issues in children with acute lymphoblastic leukemia in El Salvador. Eur J Cancer 2010;46:3280-6.

11. Viana MB, Fernandes RA, de Oliveira BM, Murao M, de Andrade Paes C, Duarte AA. Nutritional and socio-economic status in the prognosis of childhood acute lymphoblastic leukemia. Hematologica 2001;86:113-20.

12. Mostert S, Sitaresmi MN, Gundy CM, Sutaryo, Veerman AJ. Attitude of health-care providers towards childhood leukemia patients with different socio-economic status. Pediatr Blood Cancer 2008;50:1001-5.

13. McWhirter WF, Smith H, McWhirther KM. Social class as a prognostic variable in acute lymphoblastic leukemia. Med J Aust 1983;2:319-21.

14. Charalampopoulou A, Petridou E, Spyridopoulos T, et al. An integrated evaluation of socioeconomic and clinical factors in the survival from childhood acute lymphoblastic luekaemia: a study in Greece. Eur J Cancer Prevention 2004;13:397-401.

15. Schillinger JA, Grosclaude PC, Honjo S, Quinn MJ, Sloggett A, Coleman MP. Survival after acute lymphoblastic leukaemia: Effects of socioeconomic status and geographic region. Arch Dis Child 1999;80:311-7.

16. Bhatia S. Influence of race and socioeconomic status on outcome of children treated for childhood acute lymphoblastic leukemia. Curr Opin Ped 2004;16:9-14.

17. Bhatia S, Sather HN, Heerema NA, Trigg ME, Gaynon PS, Robison LL. Racial and ethnic differences in survival of children with acute lymphoblastic leukemia. Blood 2002;100:1957-64.

18. Simon C, Zyzanski SJ, Eder M, Raiz P, Kodish ED, Siminoff LA. Groups potentially at risk for making poorly informed decisions about entry into clinical trials for childhood cancer. J Clin Oncol 2003;21:2173-8.

19. Drotar D, Miller V, Willard V, Anthony K, Kodish E. Correlates of parental participation during informed consent for randomized clinical trials in the treatment of childhood leukemia. Ethics Behav 2004;14:1-15.

20. Moher D, Liberati A, Tetzlaff J, Altman DG, Group. TP. Preferred reporting items for systematic reviews and meta-analyses: The PRISMA statement. J Clin Epidmiol 2009;62:1006-12.

21. Harvey RC, Mullighan CG, Chen IM, et al. Rearrangement of CRLF2 is associated with mutation of JAK kinases, alternation if IKZF1, Hispanic/Latino ethnicity, and a poor outcome in pediatric B-progenitor acute lymphoblastic leukemia. Blood 2010;115:5312-21.

22. Hayden JA, Cote P, Bombardier C. Evaluation of the quality of prognosis studies in systematic reviews. Ann Intern Med 2006;144:427-37.

23. Bhatia S. Disparities in cancer outcomes: Lessons learned from children with cancer. Pediatr Blood Cancer 2011;56:994-1002.
