# Supplementary material for: Low Socioeconomic Status Is Associated with Worse Survival in Children with Cancer: A Systematic Review
Source: PLoS One. 2014 Feb 26;9(2):e89482. doi: 10.1371/journal.pone.0089482 (PMC3935876; doi:10.1371/journal.pone.0089482)
Supplement: Text S2 — Search Strategies. (DOCX) [file pone.0089482.s005.docx]

**Text S2. Search Strategies**

The searches were run using the OvidSP search platform in MEDLINE and EMBASE, and using the EBSCOHost search platform in CINAHL to include articles indexed as of December 10, 2012. The search strategies retrieved a total of 8,722 references. All references were saved in an EndNote library used to identify 985 duplicates. The remaining 7,737 unique references were reviewed against the inclusion criteria (see text).

The following tables record the search strategies and terms used in each of the databases.

**MEDLINE**

The search strategy for OvidSP MEDLINE (1946 to December 10, 2012) retrieved 4,533 references.

| Set | History | Results | Comments |
| --- | --- | --- | --- |
| 1 | "emigration and immigration"/ or residence characteristics/ or "catchment area (health)"/ or housing/ or public housing/ or health status disparities/ or Healthcare Disparities/ or rural health services/ or suburban health services/ or urban health services/ or exp Insurance/ or exp Health Services Accessibility/ or exp Socioeconomic Factors/ | 54,3627 | SES Terms |
| 2 | Exp Neoplasms/ | 2,416,057 | Neoplasm terms |
| 3 | 1 and 2 | 3,227,924 | Base clinical set |
| 4 | limit 3 to "all child (0 to 18 years)" | 4,042 | Age group limit |
| 5 | (infan* or child* or adolescen* or youth* or teen* or pediatric* or paediatric*).mp. | 2,961,284 | Age group textword terms |
| 6 | 4 or (3 and 5) | 4,533 | FINAL Results |

**EMBASE**

The search strategy for OvidSP EMBASE (1980 to 2012 Week 49) retrieved 3,074 references.

| Set | History | Results | Comments |
| --- | --- | --- | --- |
| 1 | exp migration/ or exp socioeconomics/ or (*housing/ or *health care disparity/ or *health disparity/ or *rural health care/ or *urban area/ or *suburban area/ or *urban population/ or *urban rural difference/ or *rural area/ or *rural population/ or exp *insurance/ or exp *health care delivery/ or *"social aspects and related phenomena"/ or *social aspect/ or exp *social status/ or exp *social structure/) | 797,225 | SES Terms |
| 2 | exp *neoplasm/ | 2,340,754 | Neoplasm terms |
| 3 | 1 and 2 | 21,716 | Base clinical set |
| 4 | limit 3 to (infant <to one year> or child <unspecified age> or preschool child <1 to 6 years> or school child <7 to 12 years> or adolescent <13 to 17 years>) | 2,483 | Age group limit |
| 5 | (infan* or child* or adolescen* or youth* or teen* or pediatric* or paediatric*).mp. | 2,809,801 | Age group textword terms |
| 6 | 4 or (3 and 5) | 3,074 | FINAL Results |

**CINAHL**

The search strategy for OvidSP EMBASE (1980 to 2012 Week 49) retrieved 3,074 references.

| Set | History | Results | Comments |
| --- | --- | --- | --- |
| S1 | MH "Socioeconomic Factors+") OR (MH "Emigration and Immigration") OR (MH "Residence Characteristics") OR (MH "Transients and Migrants") OR (MH "Relocation") OR (MH "Insurance+") OR (MH "Catchment Area (Health)") OR (MH "Public Housing") OR (MH "Housing") OR (MH "Health Status") OR (MH "Rural Health Services") OR (MH "Urban Health Services") OR (MH "Suburban Health") OR (MH "Urban Health") OR (MH "Rural Health") OR (MH "Health Services Accessibility+") | 259,664 | SES Terms |
| S2 | (MH "Neoplasms+") | 158,670 | Neoplasm terms |
| S3 | S1 and S2 | 7742 | Base clinical set |
| S4 | (MH "Child+") OR "child" OR (MH "Infant+") OR "infant" OR (MH "Adolescence") OR "adolescent" | 399580 | Age group textword terms |
| S5 | S1 AND S2 Narrow by SubjectAge: - all infant or adolescent: 13-18 years or all child | 1039 | Age Group limit |
| S6 | S5 or (S3 and S4) | 1115 | FINAL Results |
