# Supplementary material for: Low Socioeconomic Status Is Associated with Worse Survival in Children with Cancer: A Systematic Review
Source: PLoS One. 2014 Feb 26;9(2):e89482. doi: 10.1371/journal.pone.0089482 (PMC3935876; doi:10.1371/journal.pone.0089482)
Supplement: Text S3 — Data Abstraction Form. (DOCX) [file pone.0089482.s006.docx]

Study Number and First Author: Abstractor: YY XX

Year of Publication: Study dates (year only): ______________ to ______________

Country/countries of study: ________________

World Bank Status: □ LIC □ MIC □ IC

Number of pediatric (<19 years at diagnosis) patients: _________

Malignancy/malignancies: __________________________________________________________

Study design: □ RCT □ Retrospective Cohort □ Prospective Cohort □ Case-Control

□ Single centre □ Multi-centre

**Section 1 – Treatment and Outcome**

Which of the following modalities were used as part of the treatment protocol (may check more than one):

- Chemotherapy
- Radiation
- Surgery
- Transplantation

Treatment protocol name: ____________________________

What was the primary outcome?

- Overall survival (OS)
- Event free survival (EFS)
- Disease free survival (DFS)
- Treatment related mortality (TRM
- Relapse
- Abandonment of therapy

Primary outcome magnitude (including SD if reported): __________________

If time based and multiple times reported, use longest time point. Time point used: _________

**Section 2 – Socioeconomic sub groups**

Use one sheet per socioeconomic variable used (e.g. if both family income and maternal education examined as possible predictors, use one sheet for each).

Which socioeconomic variable was used: ___________________________

Was the variable individual (e.g. personal or family income) or ecologic (e.g. neighborhood income)?

□ Individual □ Ecologic

Was a definition of the socioeconomic variable provided?

- Y – *If yes, describe:*
- N

For each variable subgroup (e.g. low vs. high income), record the following if present:

| **Subgroup** | **Definiton** | **N** | **Outcome** | **Univariate (95^th^ CI)** | | | **Multivariable (95^th^ CI)** | | |
| --- | --- | --- | --- | --- | --- | --- | --- | --- | --- |
|  |  |  |  | **OR** | **RR** | **HR** | **OR** | **RR** | **HR** |
|  |  |  |  |  |  |  |  |  |  |
|  |  |  |  |  |  |  |  |  |  |
|  |  |  |  |  |  |  |  |  |  |
|  |  |  |  |  |  |  |  |  |  |

If multivariable analysis was performed, record what other variables were included in the model and the magnitude of the association

| **Variable** | **Multivariable (95^th^ CI)** | | |
| --- | --- | --- | --- |
|  | **OR** | **RR** | **HR** |
|  |  |  |  |
|  |  |  |  |
|  |  |  |  |
|  |  |  |  |
|  |  |  |  |
|  |  |  |  |
|  |  |  |  |

**Section 2 – Study Validity**

Study Participation

The source population or population of interest is adequately described for key characteristics

□ Yes □ Partly □ No □ Unsure □ Not Applicable

The sampling frame and recruitment are adequately described, including methods to identify the sample, period of recruitment and place of recruitment

□ Yes □ Partly □ No □ Unsure □ Not Applicable

Inclusion and exclusion criteria are adequately described

□ Yes □ Partly □ No □ Unsure □ Not Applicable

There is adequate participation in the study by eligible individuals

□ Yes □ Partly □ No □ Unsure □ Not Applicable

The baseline study sample is adequately described for key characteristics

□ Yes □ Partly □ No □ Unsure □ Not Applicable

**The study sample represents the population of interest on key characteristics, sufficient to limit potential bias to the results**

**□ Yes □ Partly □ No □ Unsure □ Not Applicable**

Study Attrition

Response rate (i.e. proportion of study sample completing the study and providing outcome data) is adequate

□ Yes □ Partly □ No □ Unsure □ Not Applicable

Attempts to collect information on participants who dropped out of the study are described

□ Yes □ Partly □ No □ Unsure □ Not Applicable

Reasons for loss to follow-up are provided

□ Yes □ Partly □ No □ Unsure □ Not Applicable

Participants lost to follow-up are adequately described for key characteristics

□ Yes □ Partly □ No □ Unsure □ Not Applicable

There are no important differences between key characteristics and outcomes in participants who completed the study and those who did not

□ Yes □ Partly □ No □ Unsure □ Not Applicable

**Loss to follow-up is not associated with key characteristics sufficient to limit potential bias**

**□ Yes □ Partly □ No □ Unsure □ Not Applicable**

Socioeconomic Variables Measurement

A clear definition or description of the socioeconomic variables measured is provided

□ Yes □ Partly □ No □ Unsure □ Not Applicable

Continuous variables are reported or appropriate cut-offs are used

□ Yes □ Partly □ No □ Unsure □ Not Applicable

The socioeconomic variables measures and methods are adequately valid and reliable to limit misclassification bias

□ Yes □ Partly □ No □ Unsure □ Not Applicable

Adequate proportion of the study sample has complete data for socioeconomic variables

□ Yes □ Partly □ No □ Unsure □ Not Applicable

The method and setting of measurement are the same for all study participants

□ Yes □ Partly □ No □ Unsure □ Not Applicable

Appropriate methods are used if imputation is used for missing socioeconomic data

□ Yes □ Partly □ No □ Unsure □ Not Applicable

**The socioeconomic variables of interest are adequately measured in study participants to sufficiently limit potential bias**

**□ Yes □ Partly □ No □ Unsure □ Not Applicable**

Outcome Measurement

A clear definition of the outcome is provided, including duration of follow-up

□ Yes □ Partly □ No □ Unsure □ Not Applicable

The outcome measure and method used are adequately valid and reliable to limit misclassification bias

□ Yes □ Partly □ No □ Unsure □ Not Applicable

The method and setting of measurement are the same for all study participants

□ Yes □ Partly □ No □ Unsure □ Not Applicable

**The outcome of interest are adequately measured in study participants to sufficiently limit potential bias**

**□ Yes □ Partly □ No □ Unsure □ Not Applicable**

Confounding Measurement and Account

All important confounders, including treatments are measure

□ Yes □ Partly □ No □ Unsure □ Not Applicable

Clear definitions of the important confounders measured are provided

□ Yes □ Partly □ No □ Unsure □ Not Applicable

Measurement of all important confounders is adequately valid and reliable

□ Yes □ Partly □ No □ Unsure □ Not Applicable

The method and setting of confounding measurement are the same for all study participants

□ Yes □ Partly □ No □ Unsure □ Not Applicable

Appropriate methods are used if imputation is used for missing confounder data

□ Yes □ Partly □ No □ Unsure □ Not Applicable

Important potential confounders are accounted for in the study design (e.g. matching for key variables, stratification or initial assembly of comparable groups)

□ Yes □ Partly □ No □ Unsure □ Not Applicable

Important potential confounders are accounted for in the analysis (i.e. appropriate adjustment)

□ Yes □ Partly □ No □ Unsure □ Not Applicable

**Important potential confounders are appropriately accounted for, limiting potential bias with respect to the socioeconomic variables of interest**

**□ Yes □ Partly □ No □ Unsure □ Not Applicable**

Analysis

There is sufficient presentation of data to assess the adequacy of the analysis

□ Yes □ Partly □ No □ Unsure □ Not Applicable

The strategy for model building (i.e. inclusion of variables) is appropriate and is based on a conceptual framework or model

□ Yes □ Partly □ No □ Unsure □ Not Applicable

The selected model is adequate for the design of the study

□ Yes □ Partly □ No □ Unsure □ Not Applicable

There is no selective reporting of the results

□ Yes □ Partly □ No □ Unsure □ Not Applicable

**The statistical analysis is appropriate for the design of the study, limiting potential for presentation of invalid results**

**□ Yes □ Partly □ No □ Unsure □ Not Applicable**
